# Gene regulatory network modeling via global optimization of high-order dynamic Bayesian network

**DOI:** 10.1186/1471-2105-13-131

**Published:** 2012-06-13

**Authors:** Nguyen Xuan, Madhu Chetty, Ross Coppel, Pramod P Wangikar

**Affiliations:** 1 Gippsland School of Information Technology, Monash University, Melbourne, Australia; 2 Department of Microbiology, Monash University, Melbourne, Australia; 3 Chemical Engineering Department, Indian Institute of Technology, Bombay, India

## Abstract

**Background:**

Dynamic Bayesian network (DBN) is among the mainstream approaches for modeling various biological networks, including the gene regulatory network (GRN). Most current methods for learning DBN employ either local search such as hill-climbing, or a meta stochastic global optimization framework such as genetic algorithm or simulated annealing, which are only able to locate sub-optimal solutions. Further, current DBN applications have essentially been limited to small sized networks.

**Results:**

To overcome the above difficulties, we introduce here a deterministic global optimization based DBN approach for reverse engineering genetic networks from time course gene expression data. For such DBN models that consist only of inter time slice arcs, we show that there exists a polynomial time algorithm for learning the globally optimal network structure. The proposed approach, named GlobalMIT^+^, employs the recently proposed information theoretic scoring metric named mutual information test (MIT). GlobalMIT^+^ is able to learn high-order time delayed genetic interactions, which are common to most biological systems. Evaluation of the approach using both synthetic and real data sets, including a 733 cyanobacterial gene expression data set, shows significantly improved performance over other techniques.

**Conclusions:**

Our studies demonstrate that deterministic global optimization approaches can infer large scale genetic networks.

## Background

Gene regulatory network (GRN) reverse-engineering has been a subject of intensive study within the systems biology community during the last decade. Of the dozens of methods available currently, most can be broadly classified into three main-stream categories, namely *co-expression networkdifferential equation* and *Bayesian network*. Co-expression network [1,2] is a class of coarse-scale, simplistic models that relies directly on pairwise or low-order conditional pairwise association measures, such as the (partial) correlation or (conditional) mutual information, for inferring the connectivities between genes. These methods have the advantage of low computational complexity, and can scale up to very large networks of thousands of genes [3]. However, their major limitation is that they do not model the network dynamics, and hence cannot perform prediction. Differential equation (DE) based approaches are a class of sophisticated, well established methods which have long been used for modeling biochemical phenomena, including GRNs [4,5]. A particularly salient feature of DE-based approaches is that they can accurately model the detailed dynamics of biochemical systems in continuous time. However, these methods are also much more computationally intensive, and so far are only applicable to relatively small networks of a handful genes (i.e., 5–30). Lying in-between these two extremes are Bayesian networks (BN), a class of models that are based on solid principles of probability and statistics. A BN represents accurately and compactly the joint distribution of a set of variables, using probability and graph theories. BN can also perform prediction of the GRN behavior in unknown conditions, albeit not at as detailed level as DE-based approaches.

In this paper, we focus on the BN paradigm, which is indeed among the first approaches for reverse engineering GRN, through the seminal work of Friedman et al. [6,7], and later by numerous other authors [8-14]. Two critical limitations when applying the traditional static BN paradigm to the GRN domain are: *(i)* BN does not have a mechanism for exploiting the temporal aspect of time-series data (such as time-series microarray data) abundant in this field; and *(ii)* BN does not allow the modeling of cyclic phenomena, such as feedback loops, which are prevalent in biological systems [15]. These limitations motivated the development of the dynamic Bayesian network (DBN) which has received significant interest from the bioinformatics community [15-22]. DBN exploits the temporal aspect of time series data to infer edge directions, and also allows the modeling of feedback loops (in the form of time delayed cyclic interactions).

In DBN framework, the task of GRN reverse engineering amounts to learning the optimal DBN structure from gene expression data. After the structure has been reconstructed, a set of conditional probability tables can be easily learned, using methods such as maximum likelihood, to describe the system dynamics. In this paper, we are focusing on the more challenging problem of structure learning. Most of the recent works have employed either *local search* (e.g., greedy hill climbing), *stochastic global optimization* (e.g., genetic algorithm, simulated annealing), or *Monte Carlo simulation*. This is due to several NP-hardness results for learning static BN structure (see e.g., [23]). However recently, Dojer [24] has shown otherwise that for certain DBN models, learning can be efficiently done in polynomial time for the globally optimal DBN, when the Minimum Description Length (MDL) and the Bayesian-Dirichlet equivalent (BDe) scoring metrics are employed. In our recent preliminary work [25], we have shown that this result also holds true for the Mutual Information Test (MIT), a novel scoring metric recently introduced for learning static BN [26]. Through extensive experimental evaluation, de Campos [26] suggested that MIT can compete favorably with Bayesian scores, outperform MDL (which is equivalent to the Bayesian Information Criterion—BIC) and hence should be the score of reference within those based on information theory. To our knowledge, other than the popular scoring metrics, MIT has not been considered for learning DBN. An attractive characteristic of MIT is that when placed into a global optimization framework, its complexity is much lower than that of the BDe-based algorithm by Dojer [24], and seems to be comparable to that of the MDL-based algorithm. In other words, MIT seems to combine the goodness of both BDe and MDL, namely network quality and speed. The implementation of our MIT based algorithm, made available as the GlobalMIT toolbox [27], when tested on small scale synthetic data [25], confirmed that MIT also performs competitively with BDe and MDL in terms of network quality.

The first-order Markov DBN model that we considered earlier [25,27] is however not completely adequate for the accurate modeling of GRN, as genetic interactions are invariably delayed with different time lags [20]. Specifically, this delay is due to the time required for the regulator gene to express its protein product and the transcription of the target gene to be affected (directly or indirectly) by this regulator protein. In GRNs, most genetic interactions are time delayed, depending on the time required for the translation, folding, nuclear translocation, turnover for the regulatory protein, and elongation of the target gene mRNA [28]. Furthermore, the amount of time lag needed for different regulator to exert its effect is also different. Higher order DBNs are therefore needed to capture these time-delayed interactions. In this paper, we generalize our GlobalMIT algorithm to the case of higher order DBN models, to be named GlobalMIT^+^. Our contribution in this paper is three-fold: (i) we prove the polynomial time complexity of GlobalMIT^+^ for higher order DBNs; (ii) we give a complete characterization of the time complexity of GlobalMIT^+^, and propose a variant GlobalMIT^*^ for large scale networks that balances optimality, order coverage and computational tractability; (iii) we evaluate the high-order GlobalMIT^+/*^ on several real and synthetic datasets, and for the first time apply a DBN-based GRN reverse engineering algorithm on a large scale network of 733 cyanobacterial genes, in a very reasonable run-time on a regular desktop PC. We show that the learned networks exhibit a scale-free structure, the common topology of many known biochemical networks, with hubs with significantly enriched functionals corresponding to major cellular processes.

## Methods

### Preliminaries

We first briefly review the DBN models. Let $X=\left\{X_{1},\ldots ,X_{n}\right\}$ be a set of random variables (RV); $\left\{x_{i1},\ldots ,x_{\mathrm{iN}}\right\}$ be an actual observed sequence corresponding to *X*_*i*_ over *N* time points; *X*_*i*_*t* be the RV representing the value of *X*_*i*_ at any time *t*; and $X[t]=\left\{X_{1}[t],\ldots ,X_{n}[t]\right\}$. A DBN represents the joint probability distribution function (PDF) over the set of *n*×*N* RVs X[1]∪X[2]…∪X[N]. Since such a general PDF can be arbitrarily complex, several assumptions are often employed for its simplification. The two most popular assumptions are *first-order Markovianity*, i.e., *P*(**X***t*|**X**[1],…,**X***t*−1])=*P*(**X***t*|**X***t*−1]), and *stationarity*, i.e., *P*(**X***t*|**X***t*−1]) is independent of *t*. These two assumptions give rise to the popular first-order Markov stationary DBN which assumes that both the structure of the network and the parameters characterizing it remain unchanged over time. It is worth noting that recent works have progressed to allow more flexible, non-stationary DBN models, such as ones with, either parameters [22], or both structure and parameters [29] changing over time. However, more flexible models generally require more data to be learned accurately. In situations where training data are scarce, such as in microarray experiments where the data size can be as small as a couple of dozen samples, a simpler model such as the Markov stationary DBN might be a more suitable choice.

**Figure 1:**
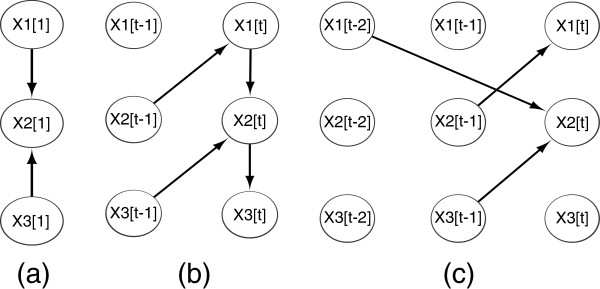
(a) prior network; (b) First-order Markov transition network; (c) 2nd-order Markov transition network with only inter time slice edges.

DBN models consist of two parts: the *prior network* and the *transition network*[30]. The prior network contains only intra time slice edges (since there are no other time slices preceding it), while the transition network can contain both inter and intra time slice edges, as demonstrated in Figure 1(a,b). Learning the prior network requires collecting *m* independent observation sequences, of which only *m* initial time slices are used for learning. For biological networks, such data abundance is not always available, since there may be only one or a very limited number of time series. Therefore, only the learning of the transition network is practical and is relevant. *Henceforth, by DBN we mean only the transition network part of the model*. Some authors have further restricted the transition network to contain only inter time slice edges [18,21,24]. In the context of genetic networks, inter-time slice edges correspond to time-delayed genetic interactions, while intra-time slice edges correspond to instantaneous interactions. In reality, only delayed genetic interactions are biologically plausible, as a result of the time required for the translation, folding, nuclear translocation, turnover time-scales for the regulatory protein, and the time scale for elongation of the target gene mRNA [28]. Only when this total time lag is small compared to the sampling gap, then the interaction can be considered as instantaneous. *In this paper we shall consider DBN with only inter-time slice edges*. The rationale for this focus can be taken from both a biological point of view (genetic interactions are essentially time-delayed), and from an algorithmic point of view: there are efficient polynomial time algorithms for learning this class of DBN, as will be discussed in the next section.

A critical limitation of the first-order DBN for modeling GRN is that it assumes every genetic interaction to have a uniform time lag of 1 time unit, i.e., all edges are from slice *t*-1] to *t*. For GRNs this is not always the case, since genetic interactions can have longer lags, and different transcription factors (TF) of the same gene can have different lags [20]. As mentioned earlier, this motivates the use of higher order DBNs, in which the *first-order Markovianity* is replaced by the *d*^th^*order Markovianity*, i.e., *P*(**X***t*|**X**[1],…,**X***t*−1])=*P*(**X***t*|**X***t*−1],…,**X***t*−*d*). With this model, a node (i.e., gene) can have parents (i.e., TFs) in any of the previous *d* time slices. A 2nd-order Markov DBN is illustrated in Figure 1(c), in which node *X*_2_ is regulated by two parents, namely *X*_3_ with one-time-unit lag, and *X*_1_ with two-time-unit lag.

### The MIT scoring metric

In this section, we first review the MIT scoring metric for learning BN and then show how it can be adapted to the DBN case. The most popular approaches for learning DBN are essentially those that have been adapted from the static BN literature, namely the *search*+*score* paradigm [15,21], and Markov Chain Monte Carlo (MCMC) simulation [18,29]. In this paper we apply the *search*+*score* approach, in which we specify a scoring function to assess the goodness-of-fit of a DBN given the data, and a search procedure to find the optimal network based on this scoring metric. While several popular scoring metrics for static BN, such as the Bayesian scores (K2, BD, BDe and BDeu), and the information theoretic scores (BIC/MDL, Akaike Information Critetion—AIC), can be adapted directly for DBNs, we focus on the Mutual Information Test (MIT), a recently introduced scoring metric for learning BN [26]. Briefly speaking, under MIT the goodness-of-fit of a network is measured by the total mutual information shared between each node and its parents, penalized by a term which quantifies the degree of statistical significance of this shared information. To understand MIT, let {_*r*1_,…,_*rn*_} be the number of discrete states corresponding to our set of RVs **X**={_*X*1_,…,_*Xn*_}, *D* denote our data set of *N* observations, *G* be a BN, and _**Pa***i*_={_*Xi*1_,…,*X*_*is*__*i*_} be the set of parents of *X*_*i*_ in *G* with corresponding {*r*_*i*1_,…,*r*_*is*__*i*_} discrete states, and _*si*_=|_**Pa***i*_|. The MIT score is defined as:

(1) $${\mathrm{SS}}_{\mathrm{MIT}}(G:D)=\;\overset{n}{\underset{i=1;{\mathrm{Pa}}_{i}\neq \emptyset }{\sum }}\;\left\{2N\;\cdot \;I(X_{i},{\mathrm{Pa}}_{i})\;-\;\overset{s_{i}}{\underset{j=1}{\sum }}\;\chi_{\alpha ,l_{i\sigma_{i}(j)}}\;\right\}\;\;,$$

where $I\left(X_{i},{\mathrm{Pa}}_{i}\right)$ is the mutual information between *X*_*i*_ and its parents as estimated from *D*. $\chi_{\alpha ,l_{\mathrm{ij}}}$ is the value such that $p\left(\chi^{2}\left(l_{\mathrm{ij}}\right)\leq \chi_{\alpha ,l_{\mathrm{ij}}}\right)=\alpha$ (the Chi-square distribution at significance level 1−*α*), and the term $l_{i\sigma_{i}(j)}$ is defined as:

(2) $$l_{i\sigma_{i}(j)}=\left\{\begin{array}{ll} (r_{i}-1)(r_{i\sigma_{i}(j)}-1)\overset{j-1}{\underset{k=1}{\prod }}r_{i\sigma_{i}(k)},\; & j=2\ldots ,s_{i}\\ (r_{i}-1)(r_{i\sigma_{i}(j)}-1),\; & j=1 \end{array}\right)$$

where $\sigma_{i}=\left\{\sigma_{i}(1),\ldots ,\sigma_{i}\left(s_{i}\right)\right\}$ is any permutation of the index set {1…_*si*_} of _**Pa***i*_, with the first variable having the greatest number of states, the second variable having the second largest number of states, and so on.

To make sense of this criterion, let us first point out that maximizing the first term in the MIT score, i.e., $\underset{i}{\sum }2N\cdot I(X_{i},{\mathrm{Pa}}_{i})$, can be shown to be equivalent to maximizing the log-likelihood criterion. However, learning BN by using the maximum likelihood principle suffers from overfitting, as the fully-connected network will always have the maximum likelihood. Likewise, for the MIT criterion, since the mutual information can always be increased by including additional variables to the parent set, i.e., $I(X_{i},{\mathrm{Pa}}_{i}\cup X_{j})\geq I(X_{i},{\mathrm{Pa}}_{i})$, the complete network will have the maximum total mutual information. Thus, there is a need to penalize the complexity of the learned network. Penalizing the log-likelihood criterion with $-\frac{1}{2}C(G)\log(N)$ gives us the BIC/MDL criteria, while -*C*(*G*) gives us the AIC criterion (where $C(G)=\overset{n}{\underset{i=1}{\sum }}(r_{i}-1)\overset{s_{i}}{\underset{j=1}{\prod }}r_{\mathrm{ij}}$ measures the network complexity). As for the MIT criterion, while the mutual information always increases when including additional variables to the parent set, the degree of statistical significance of this increment become negligible as more and more variables are added. This significance degree can be quantified based on a classical result in information theory by Kullback [31], which, in this context, can be stated as follows: under the hypothesis that *X*_*i*_ and *X*_*j*_ are conditionally independent given _**Pa***i*_ is true, the statistics 2*N*·*I*(_*Xi*__*Xj*_|_**Pa***i*_) approximates to a ^*χ*2^(*l*) distribution, with *l*=(_*ri*_−1)(_*rj*_−1)_*qi*_ degree of freedom, and _*qi*_=1 if _**Pa***i*_=*∅*, otherwise *q*_*i*_ is the total number of states of _**Pa***i*_, i.e., $q_{i}=\overset{s_{i}}{\underset{k=1}{\prod }}r_{\mathrm{ik}}$. Thus the second term in the MIT score penalizes the addition of more variables to the parent set. Roughly speaking, only variables that have the conditional mutual information shared with *X*_*i*_ given all the other variables in _**Pa***i*_ that is higher than 100*α*percent of the MI values under the null hypothesis of independence can increase the score. An important difference between MIT and the other information theoretic based metrics (BIC/MDL, AIC) is that the penalty term is applied individually and independently to each RV rather than to the network as a whole. For further details on the motivation and derivation of this scoring metric as well as an extensive comparison with BIC/MDL and BD, we refer readers to [26].

We next show how MIT can be adapted for the case of high-order DBN learning, by carefully addressing the issue of data alignment. The mutual information is now calculated between a parent set and its child at different time lags. At any time *t*>*d*, let **Pa**_*i*_={_*Xi*1_[*t*−_*δi*1_],…,*X*_*is*__*i*_[*t*−*δ*_*is*__*i*_]} be the parent set of _*Xi*_[*t*], with {_*δi*1_,…,*δ*_*is*__*i*_} be the actual regulation order corresponding to each parent. In this work, since we only consider DBN with inter time slice edges, 1≤_*δij*_≤*d*,∀*j* for a *d*-th order DBN. When the mutual information is calculated, the target node is always shifted by *d* units forward in time, while the parents are shifted forward by $\left\{d-\delta_{i1},\ldots ,d-\delta_{is_{i}}\right\}$ time units respectively. We define *I*_*s*_ as a time-delayed mutual information operator, which automatically shifts the target variable as well as all of its parents to the correct alignment.

The number of *effective observationsN*_*e*_ is therefore _*Ne*_=*N*−*d*, if we have only one time series of length *N*. If there are *m* separate time series, it is imperative that no wrong alignments occur at the transition between these time series when they are concatenated. The number of effective observations for multiple time series is $N_{e}=\overset{m}{\underset{i=1}{\sum }}N_{i}-\mathrm{md}$ where *N*_*i*_’s are the length of the time series. The MIT score for DBN is calculated as:

(3) $$S_{\mathrm{MIT}}^{\prime \prime }(G:D)\;=\;\overset{n}{\underset{i=1;{\mathrm{Pa}}_{i}\neq \emptyset }{\sum }}\;\left\{\;2N_{e}\;\cdot \;I_{s}(X_{i},{\mathrm{Pa}}_{i})\;-\;\overset{s_{i}}{\underset{j=1}{\sum }}\chi_{\alpha ,l_{i\sigma_{i}(j)}}\;\right\}\;\cdot$$

**Figure 2:**
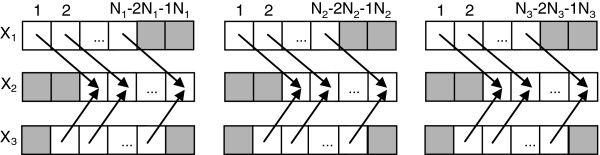
**Data alignment for node*X***_**2**_** in the DBN in Figure**1**(c).** Shaded cells denote unused observations for the calculation of *I*_*s*_(*X*_2_,Pa_2_).

To make this clear, we demonstrate the process of data alignment through the simple DBN example given in Figure 1(c). For node *X*_2_, ${\mathrm{Pa}}_{2}=\left\{X_{1}[t-2],X_{3}[t-1]\right\}$, therefore when _*Is*_(·) operates, it shifts the target node *X*_2_ forward by two units in time, while the parent *X*_1_ is shifted zero unit, and parent *X*_3_ is shifted 1 unit, as shown in Figure 2. The number of effective observations is _*Ne*_=_*N*1_−2 if only the first sequence is used, or _*Ne*_=_*N*1_ + _*N*2_ + _*N*3_−2×3 if all 3 sequences are used for learning.

### Shared and exchanged information in time-delayed MI

The proposed algorithm uses the time-delayed mutual information to give directional sense in dynamical systems. As a measure, for capturing system dynamics, the time-delayed MI contains both the exchanged information which is useful and the shared information which is not useful. However, Schreiber [32] premised that the time-delayed MI, because of its use of static probability, is limited and unable to distinguish between the exchanged information from shared information. Consequently, he proposed the concept of transfer entropy, using transition probabilities rather than static probabilities, thereby ignoring static correlations due to the common history or common input signals. From this viewpoint, it implies that the transfer entropy would be more appropriate because the time-delayed MI, using static probability, will contain exchanged information with less ‘strength’ than transfer entropy which is not influenced by static correlations.

However, we note that the transfer entropy requires the estimation of very high-dimensional joint distributions, i.e., (2*d*+1) dimensions where *d* is the Markov order. Thus, even with d = 3, hundreds to thousands of samples are required for satisfactory estimation of the 7-dimension distribution. In contrast, the time-delayed MI requires estimation of only bi-dimensional distributions and is thus better able to cope with limited (few tens of samples) microarray data samples, as commonly available for reconstructing genetic networks. If the number of samples increases in the future, e.g., due to advancements in technology for gene expression profiling, the transfer entropy approach will be an important candidate for reverse engineering genetic networks.

### Proposed approaches

This section presents our GlobalMIT^+^ algorithm for learning the globally optimal structure for a *d*-th order DBN with the MIT scoring metric in polynomial time. The original GlobalMIT algorithm for the case of the 1st-order Markov DBN [25] can be considered as a special case of GlobalMIT^+^ with d=1. Our development of GlobalMIT^+^ has made use of the same set of assumptions as proposed by [24]. While therein, the DBN learning problem is placed within a generic machine learning context, herein we are focusing our attention to the particular context of GRN modeling. Next, we list the required assumptions and discuss the associated rationales along with biological plausibility.

#### Assumption 1

(acyclicity) Examination of the graph acyclicity is not required.

This assumption is valid for DBNs with no intra time slice edges. For this class of DBN, as the edges are only directed forward in time, acyclicity is automatically satisfied. The biological implication of this assumption is that we may not be able to detect the instantaneous interactions. As stated previously, the majority of genetic interactions are time-delayed. However, if the sampling gap is large, we may consider some quick interactions as instantaneous. The effect of this constraint is that, if gene *X*_1_ regulates gene *X*_2_ almost instantly, their mutual information *I*(*X*_1_,*X*_2_) will likely be maximized when their expression profiles are in synchrony, i.e., no shifting of any of the two sequences is involved. With Assumption 1 in place, we will have to consider two time-delayed mutual information values, _*Is*_(_*X*1_,_*X*2_) and _*Is*_(_*X*2_,_*X*1_) (since *I*_*s*_ is asymmetric). If these values are significantly weaker than *I*(_*X*1_,_*X*2_) then the interaction between genes *X*_1_ and *X*_2_ may go undetected. However, when the signal is smooth and is sampled in short time step, we found that shifting the expression profile by just one time unit will not often cause a large reduction in the MI value. This is because smooth time series have high auto-correlation at short lags, and thus, instantaneous interactions may still be captured by DBN models with only inter-time slice edges. The algorithmic implication of Assumption 1 becomes clear when we consider Assumption 2 below:

#### Assumption 2

(additivity) $S(G:D)=\overset{n}{\underset{i=1}{\sum }}s(X_{i},{\mathrm{Pa}}_{i}:D{|}_{X_{i}\cup {\mathrm{Pa}}_{i}})$ where $D{|}_{X_{i}\cup {\mathrm{Pa}}_{i}}$ denotes the restriction of *D* to the values of the members of $X_{i}\cup {\mathrm{Pa}}_{i}$.

To simplify notation, we write *s*(_**Pa***i*_) for $s\left(X_{i},{\mathrm{Pa}}_{i}:D{|}_{X_{i}}\cup {\mathrm{Pa}}_{i}\right)$. Assumption 2 simply states that the scoring function decomposes over the variables and is satisfied by most scoring metrics such as BIC/MDL, BD and also clearly by MIT. However together with Assumption 1, their algorithmic implication is profound: these assumptions allow us to compute the parent set for each node independently. Unlike the case of BN where the choice of parents for a certain node may affect the choice of parents of all the other nodes, for DBN (without intra time slice edges), the choice of parents for a node has no effect on the other nodes. Thus, the algorithms developed based upon these two assumptions become very amenable to parallelization, i.e., each node can be learned independently with a separate computational thread. Still, exhaustive brute-force search for the optimal parent set will require exponential time for a *d*-th order DBN, because _**Pa***i*_ can be an arbitrary subset of X[t−1]∪…∪X[t−d] and the number of all possible parent sets is 2^dn^. In order to further reduce the search space, we rely on the special structure of the scoring metric, as follows:

#### Assumption 3

(splitting) *s*(_**Pa***i*_)=*u*(_**Pa***i*_) + *v*(_**Pa***i*_) for some non-negative functions *u* and *v* satisfying ${\mathrm{Pa}}_{i}\subseteq {\mathrm{Pa}}_{i}^{\prime \prime }\Rightarrow u({\mathrm{Pa}}_{i})\leq u({\mathrm{Pa}}_{i}^{\prime \prime })$.

#### Assumption 4

(uniformity) $|{\mathrm{Pa}}_{i}|=|{\mathrm{Pa}}_{i}^{\prime \prime }|\Rightarrow u({\mathrm{Pa}}_{i})=u({\mathrm{Pa}}_{i}^{\prime \prime }).$

Assumption 3 requires the scoring function to decompose into two components: *v* evaluating the accuracy of representing the distribution underlying the data by the network, and *u* measuring its complexity. Furthermore, *u* is required to be a monotonically non-decreasing function in the cardinality of **Pa**_*i*_ (Assumption 4), i.e., the network gets more complex as more variables are added to the parent sets. However in its original form, the MIT scoring metric, having higher scores for better networks, does not abide by these assumptions. We overcome this by casting the problem as a minimization problem (similar to Dojer) where lower scored networks are better. We consider a variant of MIT as follows:

(4) $$\;S_{\mathrm{MIT}}(G:D)\;=\;\overset{n}{\underset{i=1}{\sum }}2N_{e}\cdot I_{s}(X_{i},X^{d})\;-\;S_{\mathrm{MIT}}^{\prime \prime }(G\;:\;D),$$

where $X^{d}=X[t-1]\cup \ldots \cup X[t-d]$. This score admits the following decomposition over each variable (with the convention of *I*(_*Xi*_,*∅*)=0):

(5) $$\begin{array}{lcrr} s_{\mathrm{MIT}}({\mathrm{Pa}}_{i}) & = & v_{\mathrm{MIT}}({\mathrm{Pa}}_{i})+u_{\mathrm{MIT}}({\mathrm{Pa}}_{i}), & \\ v_{\mathrm{MIT}}({\mathrm{Pa}}_{i}) & = & 2N_{e}\cdot I_{s}(X_{i},X^{d})-2N_{e}\cdot I_{s}(X_{i},{\mathrm{Pa}}_{i}), & \\ u_{\mathrm{MIT}}({\mathrm{Pa}}_{i}) & = & \overset{s_{i}}{\underset{j=1}{\sum }}\chi_{\alpha ,l_{i\sigma_{i}(j)}}. & \end{array}$$

Roughly speaking, *v*_*MIT*_ measures the “error” of representing the joint distribution underlying *D* by *G*, while *u*_*MIT*_ measures the complexity of this representation. We make the following propositions:

#### Proposition 1

*S’*_*MIT*_ maximization is equivalent to *S*_*MIT*_ minimization.

#### Proof

This is obvious, since $\overset{n}{\underset{i=1}{\sum }}2N_{e}\cdot I_{s}(X_{i},{\text{X}}^{d})=\mathrm{constant}$. □

#### Proposition 2

_*vMIT*_,_*uMIT*_satisfy assumption 3.

#### Proof

_*vMIT*_≥0 since of all possible parent sets _**Pa***i*_, the full set ^**X***d*^has the maximum (shifted) mutual information with *X*_*i*_. And since the support of the Chi-square distribution is ^*ℝ* + ^, i.e., _*χα*,·_≥0, therefore ${\mathrm{Pa}}_{i}\subseteq {\mathrm{Pa}}_{i}^{\prime \prime }\Rightarrow 0\leq u_{\mathrm{MIT}}({\mathrm{Pa}}_{i})\leq u_{\mathrm{MIT}}({\mathrm{Pa}}_{i}^{\prime \prime })$. □

While we note that *u*_*MIT*_ does not satisfy Assumption 4, for applications where all the variables have the same number of states, it can be shown to satisfy this assumption. Within the context of GRN modeling from microarray data, this generally holds true, since it is a popular practice to discretize expression data of all genes to, e.g., 3 states corresponding to high, low and base-line expression value [15].

#### Assumption 5

(variable uniformity) All variables in **X** have the same number of discrete states *k*.

#### Proposition 3

Under the assumption of variable uniformity, *u*_*MIT*_ satisfies assumption 4.

#### Proof

It can be seen that if |**Pa**_*i*_|=|**Pa**_*i*_*″*|=_*si*_, then $u_{\mathrm{MIT}}({\mathrm{Pa}}_{i})=u_{\mathrm{MIT}}({\mathrm{Pa}}_{i}^{\prime \prime })=\overset{s_{i}}{\underset{j=1}{\sum }}\chi_{\alpha ,{\left(k-1\right)}^{2}k^{j-1}}$. □

Since _*uMIT*_(_**Pa***i*_) is the same for all parent sets of the same cardinality, we can write _*uMIT*_(|_**Pa***i*_|) in place of _*uMIT*_(_**Pa***i*_). With Assumptions 1-5 satisfied, we can employ the following Algorithm 1, named globalMIT^+^, to find the globally optimal DBN with MIT, i.e., the one with the minimal *S*_*MIT*_ score.

#### Theorem 1

Under assumptions 1-5, GlobalMIT^+^ applied to each variable in **X** finds a globally optimal *d*-th order DBN under the MIT score.

### Algorithm 1 GlobalMIT^+^ : Optimal *d*^th^-order DBN with MIT

· _**Pa***i*_:=*∅*

· **for***p*=1 to *nd*

· If _*uMIT*_(*p*)≥_*sMIT*_(_**Pa***i*_) then return _**Pa***i*_; Stop.

· $P=\text{arg min}\left\{s_{\mathrm{MIT}}(\text{Y})|\text{Y}\subseteq X^{d};|\text{Y}|=p\right\}$

· If _*sMIT*_(**P**)<_*sMIT*_(_**Pa***i*_) then _**Pa***i*_:=**P**.

· **end for**

#### Proof

The key point here is that once a parent set grows to a certain extent, its complexity alone surpasses the total score of a previously found sub-optimal parent set. In fact, all the remaining potential parent sets **P** omitted by the algorithm have a total score higher than the current best score, i.e., _*sMIT*_(**Pa**)≥_*uMIT*_(|**Pa**|)≥_*sMIT*_(_**Pa***i*_), where _**Pa***i*_is the last sub-optimal parent set found. □

We note that the terms $2N_{e}\cdot I_{s}(X_{i},X^{d})$ in the *S*_*MIT*_ score in (1) are all constant and would not affect the outcome of our optimization problem. Knowing their exact value is however, necessary for the stopping criterion in Algorithm 1, and also for determining its complexity bound, as will be shown in Section “Complexity analysis”. Calculating $I_{s}(X_{i},X^{d})$ is by itself a hard problem, requiring in general, a space and time complexity of order *O*(^*knd* + 1^). However, for our purpose, since the only requirement for *v*_*MIT*_ is that it must be non-negative, it is sufficient to use an upper bound of $I_{s}(X_{i},X^{d})$. Since a fundamental property of the mutual information states that I(U,V)≤min{H(U),H(V)}, i.e., mutual information is bounded by the corresponding entropies, we have:

(6) $$2N_{e}\cdot I_{s}(X_{i},X^{d})\leq 2N_{e}\cdot H_{s}(X_{i}),$$

where _*Hs*_(_*Xi*_) is the entropy of *X*_*i*_ estimated from a *d*-time-unit shifted expression profile, i.e., $\left\{x_{i(d+1)},\ldots ,x_{\mathrm{iN}}\right\}$. Otherwise, we can use a universally fixed upper bound for all _*Hs*_(_*Xi*_), that is logk, then:

(7) $$2N_{e}\cdot I_{s}(X_{i},X^{d})\leq 2N_{e}\cdot \log\mathrm{k.}$$

Using these bounds, we obtain the following more practical versions of *d*_*MIT*_:

(8) $$\begin{array}{lcrr} v_{\mathrm{MIT}}^{\prime \prime }({\mathrm{Pa}}_{i}) & = & 2N_{e}\cdot H_{s}(X_{i})-2N_{e}\cdot I_{s}(X_{i},{\mathrm{Pa}}_{i}) & \\ v_{\mathrm{MIT}}^{\prime }({\mathrm{Pa}}_{i}) & = & 2N_{e}\cdot \log k-2N_{e}\cdot I_{s}(X_{i},{\mathrm{Pa}}_{i}). & \end{array}$$

It is straightforward to show that Algorithm 1 and Theorem 1 are still valid when *v’*_*MIT*_ or *v”*_*MIT*_ are used in place of *v*_*MIT*_.

### Complexity analysis

#### Theorem 2

GlobalMIT^+^ admits a polynomial worst-case time complexity of $O({\left(\mathrm{nd}\right)}^{\underset{k}{\log}N_{e}})$ in the number of variables and DBN order.

#### Proof

Our aim is to find a number p^*^ satisfying $u_{\mathrm{MIT}}(p^{*})\geq s_{\mathrm{MIT}}(\emptyset )$. Clearly, there is no need to examine any parent set of cardinality p^*^ and over. In the worst case, our algorithm will have to examine all the possible parent sets of cardinality from 1 to p^*^-1. We have:

(9) $$\begin{array}{cc} u_{\mathrm{MIT}}(p^{*}) & \geq s_{\mathrm{MIT}}(\emptyset )\Leftrightarrow \overset{p^{*}}{\underset{j=1}{\sum }}\chi_{\alpha ,l_{i}\sigma_{i}(j)}\geq v_{\mathrm{MIT}}(\emptyset )\\ =2N_{e}\cdot I_{s}(X_{i},X^{d}). \end{array}$$

As discussed above, since calculating *v*_*MIT*_ is not convenient, we use *v’*_*MIT*_ and *v”*_*MIT*_ instead. With *v’*_*MIT*_, p^*^ can be found as:

(10) $$p^{*}=\text{arg min}\left\{p|\overset{p}{\underset{j=1}{\sum }}\chi_{\alpha ,l_{i}\sigma_{i}(j)}\geq 2N_{e}\cdot H_{s}(X_{i})\right\},$$

while for *v*_*MIT*_′:

(11) $$p^{*}=\text{arg min}\left\{p|\overset{p}{\underset{j=1}{\sum }}\chi_{\alpha ,l_{i}\sigma_{i}(j)}\geq 2N_{e}\cdot \log k\right\}.$$

It can be seen that p^*^ depends only on *α*,*k*and *N*_*e*_. Since there are $O({\left(\mathrm{nd}\right)}^{p^{*}})$ subsets of ^**X***d*^ with at most p^*^ parents, and each set of parents can be scored in polynomial time, GlobalMIT^+^ admits an overall polynomial worst-case time complexity in the number of variables *n* and network order *d*. While p^*^ does not admit a closed-form solution (since $\chi_{\alpha ,l_{\mathrm{ij}}}$ cannot be analytically calculated), a large over-estimate of p^*^ can be provided as follows. Note that $\chi_{\alpha ,l_{\mathrm{ij}}}$ is the value such that $p(\chi^{2}(l_{\mathrm{ij}})\leq \chi_{\alpha ,l_{\mathrm{ij}}})=\alpha$. Since generally *α*≫0.5, if we take the mean value (corresponding roughly to *α*=0.5) of the $\chi^{2}(l_{\mathrm{ij}})$ distribution, i.e., *l*_*ij*_, as an under-estimate for $\chi_{\alpha ,l_{\mathrm{ij}}}$, then:

(12) $$\;\begin{array}{cc} \overset{p^{*}}{\underset{j=1}{\sum }}\chi_{\alpha ,l_{i}\sigma_{i}(j)} & \geq 2N_{e}\cdot \log k\Leftrightarrow \overset{p^{*}-1}{\underset{j=0}{\sum }}{\left(k-1\right)}^{2}k^{j}\\ \geq 2N_{e}\cdot \log k\\ \Leftrightarrow (k-1)\left(k^{p^{*}}-1\right) & \geq 2N_{e}\cdot \log k\Leftrightarrow p^{*}\\ \geq \underset{k}{\log}\left(\frac{2N_{e}\cdot \log k}{k-1}+1\right) \end{array}$$

Assuming $N_{e}\gg \log k$, we can see that $p^{*}∼\underset{k}{\log}(N_{e})$, and the algorithm admits an overall complexity of $O({\left(\mathrm{nd}\right)}^{\underset{k}{\log}N_{e}})$. □

Let us now compare this bound with those of the algorithms for learning the globally optimal DBN under the BIC/MDL and BDe scoring metrics as proposed by [24], and implemented in the BNFinder software [21]. For BIC/MDL, $p_{\mathrm{MDL}}^{*}$ is given by $\left\lceil \underset{k}{\log}N_{e}\right\rceil$, while for BDe, $p_{\mathrm{BDe}}^{*}=\lceil N_{e}\underset{\lambda^{-1}}{\log}k\rceil$, where the distribution $P(G)\propto \lambda^{\sum |{\mathrm{Pa}}_{i}|}$, with a penalty parameter 0<*λ*<1, is used as a prior over the network structures [24], default value $\log\lambda^{-1}=1$ for BNFinder]. In general, $p_{\mathrm{BDe}}^{*}$ scales linearly with the number of effective data items _*Ne*_, making its value less of practical interest, even for small data sets. Moreover, this bound becomes meaningless when _*Ne*_>*n*, as $p_{\mathrm{BDe}}^{*}>n$, meaning that in the worst case BNFinder+BDe will have to investigate all the possible parent sets. On the other hand, it can be seen that the first order GlobalMIT and BNFinder+MDL admits roughly the same worst-case computational complexity.

### The GlobalMIT^*^ algorithm

It is noted that the search space has been expanded from **X**[*t*−1] in the case of the 1st-order DBN, to $X^{d}=X[t-1]\cup \ldots \cup X[t-d]$ for the case of the *d*^th^-order DBN. Roughly, the number of variables has been multiplied *d* times in order to accommodate the higher-order regulations. Such a multiplicative expansion in the search space may be very expensive, especially for a deterministic global optimization algorithm such as GlobalMIT^+^. For very large networks, it may be useful to consider the following additional assumption:

#### Assumption 6

(non-redundant, optimal-lag interaction) No multiple edges with different time lags exist between a parent *X*_*i*_ and its child *X*_*j*_. Furthermore, the only one edge allowed, if it exists, must take place at the optimal lag $\delta_{\mathrm{ij}}^{*}$, where $\delta_{\mathrm{ij}}^{*}=\text{argmax}\left\{I_{s}(X_{j},X_{i}[t-\delta ])|1\leq \delta \leq d\right\}$.

This assumption restricts that for each node *X*_*i*_, there may be only one single link to any node *X*_*j*_ at the *most-probable* time lag where their mutual information is maximized. With this assumption in place, the search space for each variable *X*_*j*_ reduces from $X^{d}=X[t-1]\cup \ldots \cup X[t-d]$ to $X_{j}^{*}={\left\{X\left[t-\delta_{\mathrm{ij}}^{*}\right]\right\}}_{i=1\ldots n}$, which is equivalent in size to the first-order GlobalMIT algorithm. Thus *Assumption 6 provides a trade-off between optimality and coverage*: while the search is performed only on *n* variables at a pre-determined lag thereby significantly reducing the computational cost, this lag can take any value from 1 to *d* detecting delayed genetic interactions at the most likely time lag. We shall refer to this variant of GlobalMIT^+^, when Assumption 6 is employed, as GlobalMIT^*^. It can be easily seen that, for any high order *d*>1, GlobalMIT^*^ still admits the same complexity as the first order GlobalMIT.

## Results and discussion

This section presents the experimental evaluation on GlobalMIT^+/*^. Our proposed algorithms are implemented within the Matlab/C++ GlobalMIT^+^ toolbox, freely available as online supplementary material (Additional file 1). We compare our approach with two other global optimization algorithms for learning DBN under the MDL and BDe metrics, namely BNFinder+MDL and BNFinder+BDe, which are part of the Python-based BNFinder software [21]. As elaborated in the previous section, the BNFinder+BDe algorithm is generally very expensive, and hence not feasible for large or even medium (few tens of nodes) scale networks. In these cases, we replace BNFinder+BDe with BANJO [33], a Java-based software package for learning DBN using the BDe metric via a stochastic global optimization method, in particular simulated annealing.

It is noted that the GlobalMIT^+^ toolbox supports multi-threading to maximally exploit the currently popular multi-core PC systems. We conducted our experiments on a quad-core i7 desktop PC with 8Gb of main memory, running Win7 64bit, which is a typical off-the-shelf PC configuration at the time this paper was written. Intel core i7 processors contain 4 separate cores, each can handle 2 independent threads concurrently. We shall execute GlobalMIT^+^ with 6 threads in parallel (the remaining two being reserved for system and interface processes). BANJO also supports multi-threading, whereas BNFinder does not. While we could have run all algorithms with only a single thread, for a “fair” comparison in terms of run-time, our objective in carrying out the experiments this way is to highlight the capability and benefit of parallelization of GlobalMIT^+^. The 1-thread execution time would be roughly three to five times longer in our observation. As for parameter setting, BNFinder was run with default settings, while BANJO was run with 6 threads, *simulated annealing+random move* as the search engine, and its run-time was set to, either that required by GlobalMIT^+^ or at least 10 minutes, whichever longer. GlobalMIT^+^ has two parameters, namely the significance level *α*, to control the trade-off between goodness-of-fit and network complexity, and the DBN order *d*. Adjusting *α* will affect the sensitivity and precision of the discovered network, very much like its affect on the Type-I and Type-II error of the mutual information test of independence. De Campos [26] suggested using high significance levels, i.e., between 0.999 and 0.9999. We note that for smaller number of samples, a lower level of significance *α*may be necessary to avoid overly penalizing network complexity. Thus, in our experiments we set *α*=0.999 for _*Ne*_<100 and *α*=0.9999 otherwise. The choice of a suitable DBN order *d*, on the other hand, is both species-specific and data-specific, in particular the data sampling rate. For example, in mammals, the transcriptional regulatory time delay can be from several minutes to several tens of minutes, and is composed of two components: the TF translation/post-translational processing/translocation time (∼10.5±4 mins), and the target gene transcription and post-transcription processing time (∼20−40 mins) [28]. Also, for a higher data sampling rate, a higher *d* value is needed to cover the same time delay. It is also noted that increasing *d* will decrease the number of effective data points available for learning. In our experiments, we experimentally set *d* from 1 to several time units, depending upon the sampling rate. Whenever necessary, gene expression data were discretized using 3-state quantile discretization.

### Small scale *E. Coli* network

We study the *E. coli* SOS system [34] which involves *lexArecA* and more than 30 other genes they directly regulate. In normal condition, *LexA* binds to the promoter regions of these genes and acts as a master repressor. When the DNA is damaged, the *RecA* protein senses the damage and triggers *LexA* autocleavage. Drop in *LexA* level leads to de-repression of the SOS genes. When DNA repair completes, *RecA* stops mediating *LexA* autocleavage, *LexA* accumulates and represses the SOS genes again. We used the expression data gathered in [34] for 8 genes, namely *uvrD, lexA, umuD, recA, uvrA, uvrY, ruvA* and *polB*, to reconstruct the interactions between these genes. The data set contains 4 time series, each of 50 observations taken at 6-minute interval, under two UV exposition levels. Since the dynamics of each gene in all time series are similar, we can take the mean value of these time series as input to the algorithms. Thus, the input data consists of 8 genes×50 observations.

**Figure 3:**
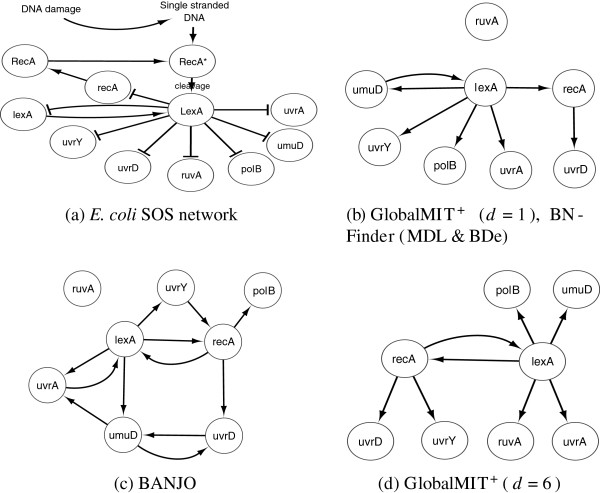
**Experimental results on the*E. coli*SOS network.**

For this small network, GlobalMIT^+^ and BNFinder require only a few seconds, while BANJO was executed for 10 minutes with 6 threads in parallel. The experimental results are reported in Figure 3. GlobalMIT^+^ (d=1), BNFinder (BDe & MDL) all returned the same network in Figure 3(b), with *ruvA* being disconnected. Overall, this structure closely reflects the SOS network, in which the *lexA/recA* compound acts as a hub that controls the other genes. BANJO returned the network in Figure 3(c), in which the hub-structure is basically also identified, but with several more false interactions between the target genes, e.g., between *umuD* and *uvrD/uvrA*. Note that the *ruvA* gene is also disconnected in the BANJO’s recovered network. When testing with higher orders, GlobalMIT^+^ discovered a similar hub structure. The most complete network was discovered at d=6 in (Figure 3d), in which all the interactions between *lexA/recA* and other genes were recovered. Furthermore, the mutual interaction between *lexA* and *recA* were also correctly identified. Additional experiments to test the effect of data discretization on this data set are presented in the online supplementary material (Additional File 2).

### Medium scale synthetic network for glucose homeostasis

**Figure 4:**
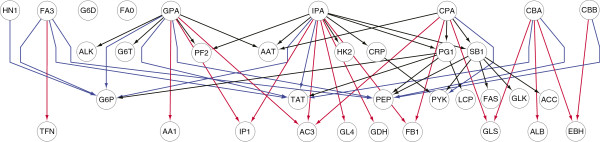
The hepatic glucose homeostasis network: black, blue, red colors for 1st-, 2nd- and 3rd-order interactions respectively.

We study a glucose homeostasis network of 35 genes and 52 interactions, first proposed by Le et al. [35]. The network, which shows the genetic interactions that control glucose metabolism in perinatal hypatocytes, was the result of an extensive literature review of the biological components affecting perinatal glucose metabolism. Le et al. [35] modeled the interactions using conditional probability tables with two discrete states, with the strength of the interactions chosen to be consistent with biological variation. They provided a program to generate synthetic data sets from this network using a *static* Bayesian network model. It is clear from Figure 4 that the network has a cascade hierarchical structure, and is reasonably complex, with several genes being regulated by multiple transcription factors. In order to create a synthetic *dynamic* Bayesian network for testing, we modified Le *et al*.’s network as follows. First, we organized the nodes into 4 levels, with the top level comprising of the master transcription factors (TFs), and the interaction order between nodes in adjacent levels assumed to be one. The network in Figure 4 thus contains time-delayed interactions of orders 1 (13 edges), 2 (23 edges) and 3 (16 edges). Then, from the data generated by Le *et al*.’s program, we simply shifted forward the expression profiles of the 2nd-, 3rd- and 4th-level nodes by 1, 2 and 3 time units respectively to create data for this DBN model. We generated ten time series of 125 observations, then for each *N*∈{25,50,75,100,125} we took the first *N* observations of these series for testing. Since the network structure in this experiment is known in advance by design, we can calculate the true positive (TP), false positive (FP) and false negative (FN) edges. The mean±standard deviation values for the performance metrics, namely *sensitivity* (=TP/(TP+FN)), *precision* (=TP/(TP+FP)) and *runtime*, over 10 time series for all algorithms are reported in Table 1.

**Table 1:** Experimental results for the hepatic glucose homeostasis network

															
	**GlobalMIT(*d=*1)**	GlobalMIT^*^(*d=*3)	**GlobalMIT**^**+**^**(*d=*3)**	**BANJO**	**BNFinder+MDL**										
**N**	**Pr**	**Se**	**Time**	**Pr**	**Se**	**Time**	**Pr**	**Se**	**Time**	**Pr**	**Se**	**Time**	**Pr**	**Se**	**Time**
25	75±17	9±2	0±0	67±8	18±5	0±0	64±12	18±5	0±0	12±2	22±3	10±0	64±17	9±2	0±0
50	82±14	19±3	0±0	80±10	35±3	0±0	77±12	35±4	0±0	25±5	27±6	10±0	88±12	18±4	1±0
75	85±12	24±3	0±0	85±6	45±4	0±0	81±8	46±4	9±0	34±4	28±2	10±0	85±11	23±3	7±0
100	94±7	24±2	2±0	98±4	46±4	0±0	98±4	46±4	11±0	41±5	29±3	11±0	85±8	25±3	14±0
125	91±8	25±2	2±0	97±4	50±3	2±0	97±4	50±4	482±39	43±4	30±3	482±39	82±8	27±2	20±0

Se: percent sensitivity; Pr: percent precision; Time: in minutes.

It is noted that we have omitted BNFinder+BDe in this experiment. The reason is that this algorithm becomes too expensive even for this medium network. For example, at N=25, BNFinder+BDe requires around 1 minute. The execution time quickly increase to 1206±167 mins at N=50. And at N=75, we could not even complete analyzing the first of the 10 datasets: the execution was abandoned after 3 days, with BNFinder+BDe having learnt the parents for only 2 nodes. Of the algorithms reported in Table 1, GlobalMIT, BANJO and BNFinder+MDL are limited to learning the 1st-order DBN. It can be observed that GlobalMIT and BNFinder+MDL learned networks with similar sensitivity and precision, with both performance metrics improving as *N* increased. On the other hand, BANJO achieved a slightly better sensitivity, but at the cost of a significantly lower precision. This observation is in concordance with our earlier experiment on the *E. coli* SOS network, in which BANJO also learned many more edges than GlobalMIT^+^ and BNFinder. This result also highlights the major advantage of deterministic global optimization based approaches (GlobalMIT^+^, BNFinder) over stochastic global optimization based method such as BANJO. Wherever applicable, these methods never get stuck in local minima, and are able to deliver consistent and high quality results. Of course, BANJO on the other hand is the choice for very large datasets where deterministic methods are computationally infeasible.

As for higher-order DBN learning algorithms, both GlobalMIT^+^ and GlobalMIT^*^ (with d=3) achieves significantly better sensitivity compared to first-order DBN learning algorithms (GlobalMIT, BNFinder, BANJO). The improved sensitivity is mainly credited to the ability of these algorithms to cover all the possible time-delayed interactions between the genes. More specifically, at N=125, GlobalMIT^*^ discovers on average 16.9 high-order interactions, i.e., 43% of the total high-order interactions. Meanwhile, BANJO and BNFinder+MDL only recover on average 5.5 (14%) and 4.6 (12%) high-order interactions respectively. It is also noticeable from this experiment that GlobalMIT^*^ delivered results almost identical to GlobalMIT^+^ but with a much shorter time, comparable to the 1st-order GlobalMIT.

### Large scale cyanobacterial genetic networks

This section presents our analysis on a large scale cyanobacterial network. Cyanobacteria are the only prokaryotes that are capable of photosynthesis, and in recent years have received increasing interest [36], due to their high efficiency in carbon sequestration and potential for biofuel production (up to 30 times more efficient than terrestrial oilseed crops). These organisms therefore are credited with holding the key to solving two of the most critical problems of our time, namely climate change and the dwindling fossil fuel reserves. Despite their evolutionary and environmental importance, the study of cyanobacteria using modern high throughput tools and computational techniques has somewhat lagged behind other model organisms. Herein, we focus on *Cyanothece* sp. 51142, hereafter *Cyanothece*, a unicellular cyanobacterial strain that is involved not only in photosynthesis but also in nitrogen fixation in the same cell. As a byproduct of nitrogen fixation, *Cyanothece* has been recently shown to produce biohydrogen at very high rates that are several fold higher than previously described hydrogen-producing photosynthetic microbes [37].

**Figure 5:**
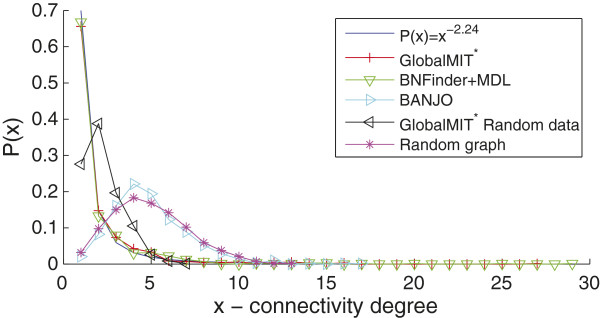
**The*Cyanothece*sp. 51142 reconstructed genetic networks, visualized with Cytoscape.** Node size is proportional to the node connectivity.

We used transcriptomic data from [36], where samples from cells grown in alternating 12h light-dark cycles were collected every 4h over a 48h time course. We analyze the subset of 733 genes that have a 2-fold expression in at least one of the 12 time points, as published in [36]. Since the sampling gap of 4h in this experiment is relatively large as compared to regular biological regulatory time lag, we used spline interpolation to interpolate two more data points in between each two actual measurements, i.e., upsampling the given time series at an 1h20’ interval. The resulting data set thus contains 733 genes×34 time points. For this large network, we employed the GlobalMIT^*^ version, with order d=3 (which indeed covers one time point lag on the original data set). GlobalMIT^*^ inferred the network as in Figure 5(a) after 14.5 mins of execution time. Upon visualization with Cytoscape [38] using a standard layout algorithm, the network shows a clear scale-free topology, with the majority of nodes having only a few connections and a small number of hubs having many connections. The node degree in a scale-free network distributes according to a power-law distribution, $P(x)\propto x^{-\gamma }$, with the scaling parameter *γ*typically between 2 and 3 for various networks in nature, society and technology. The scale-free property is thought to be a key organization feature of cellular networks, as supported by recent analysis on model organisms such as *S. cerevisiae* and *C. elegans*[39,40]. It is noted that some authors use the scale-free property as the prior input for their algorithms to, either encourage or enforce them to produce scale-free networks as output [40,41]. Herein however, we have not used any such prior information.

**Figure 6:**
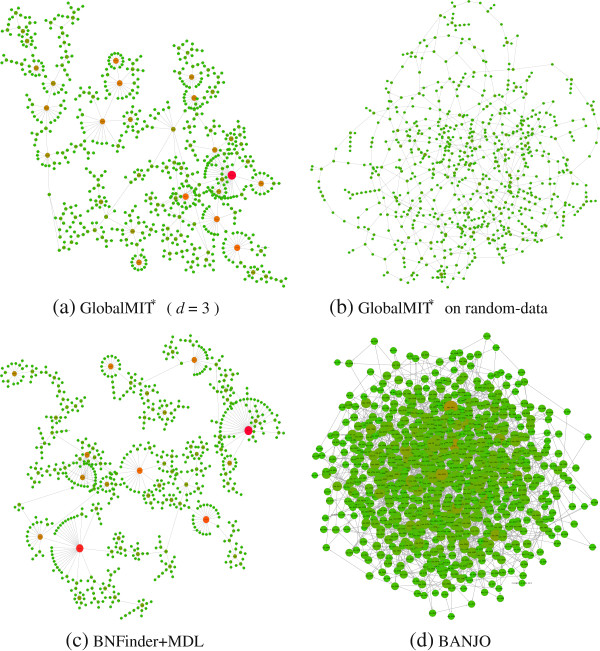
Node degree distribution.

To formalize this observation, we fit the node degree (counting both in- and out-degree) in the GlobalMIT^*^ inferred network to the power-law distribution using the method of maximum likelihood (ML). The ML estimate for *γ* in this network is 2.24, falling well within the typical range. From Figure 6 it can be seen that the observed degree distribution fits well with the theoretical *P*(*x*)=^*x*−2.24^curve. In order to verify that the scale-free structure is not merely an artefact of the inference algorithm, we test GlobalMIT^*^ with the same parameters on the same microarray data set, but with every gene expression profile randomly shuffled. The resulting network is shown in Figure 5(b). Using the same layout algorithm, no clear modular structure and hubs are visually recognizable for this network. Also, as clear from Figure 6, the node degree distribution largely deviates from a power-law curve, being very short-tailed with the largest hubs having only 7 connections.

We next tested BNFinder and BANJO on this data set. BNFinder+BDE was abandoned after 3 days of execution without finishing. BNFinder+MDL on the other hand is relatively fast, requiring only 4 mins. The resulting network, shown in Figure 5(c), also exhibits a scale-free structure. The ML estimate for *γ* in this network is, interestingly, 2.25, very close to that of the GlobalMIT^*^ network. BANJO was run with 6 threads for 1h. The resulting network, shown in Figure 5(d), does not appear to possess a scale-free topology, and the node degree distribution also largely deviates from a power-law curve. In fact, the BANJO network node degree distribution resembles that of a random Erdös-Rényi graph with the same number of nodes and connections (Figure 6).

We next perform functional enrichment analysis for the top hubs in each network. For this purpose, we gathered annotation data for *Cyanothece* sp. 51142 from Cyanobase [42, access May 2011]. Cyanobacteria in general and *Cyanothece* in particular are not very well annotated. For example, to date, nearly half of *Synechocystis* sp. PCC 6803’s genes, the best studied cyanobacterium, remain unannotated. Therefore, we supplemented Cyanobase annotation with homology search using the Blast2GO software suit [43]. In total, these combined efforts gave us annotation data for 542 out of 733 genes in our study. We then employed BiNGO [44] for gene ontology functional category enrichment analysis, using the hypergeometric test for functional over-representation, and False Discovery Rate (FDR) as the multiple hypothesis testing correction scheme. Only a corrected *p*-value of less than 0.05 is considered significant. Following these procedures, of the top 20 hubs in the GlobalMIT^*^ network, 10 were found to be significantly enriched in major *Cyanothece* cellular processes, such as nitrogen fixation, photosynthesis and other closely related pathways, as presented in Table 2. Since the wet-lab experimental setting herein involves alternative light-dark cycles, this result is found to be highly biologically relevant. *Cyanothece* strains thrive in marine environments, and in addition to carbon fixation through photosynthesis, these bacteria can also perform nitrogen fixation by reducing atmospheric dinitrogen to ammonia. Since the nitrogenase enzyme is highly sensitive to oxygen, *Cyanothece* temporally separates these processes within the same cell, so that oxygenic photosynthesis occurs during the day and nitrogen fixation during the night [36]. Thus, under normal growth condition with regular dark-light cycles and without any stress, it could be expected that photosynthesis and nitrogen fixation are the two most active *Cyanothece* cellular processes. This is reflected clearly in the GlobalMIT^*^ reconstructed network. Upon inspecting BNFinder+MDL network, 6 out of the top 20 hubs were found to be significantly enriched, also in major relevant cellular processes. It is noted that while GlobalMIT^*^ show the most hubs, BNFinder+MDL manages to recover several hubs with significantly better corrected p-value. In particular, 3 hubs for nitrogen fixation, proton transport and ribosome were recovered with significantly smaller corrected *p*-value. However, as opposed to GlobalMIT^*^, other important functional hubs for photosynthesis, photosystem I & II were missing. BANJO on the other hand produced relatively poor result, with only 1 out of 20 top hubs turned out to be significantly enriched, but not related to any major cellular pathway. The overall results suggest that both GlobalMIT^*^ and BNFinder+MDL successfully reconstructed biologically plausible network structures, i.e., scale-free with a reasonable scaling parameter value, and with functionally enriched modules relevant to the wet-lab experimental condition under study. GlobalMIT^*^ managed to produce more enriched hubs, as a result of the higher order DBN model employed and the improved MIT scoring metric. BANJO on the other hand, generally failed to produce a plausible network structure. This experimental result thus highlights the advantage of deterministic global optimization approach, as employed by GlobalMIT^*^ and BNFinder+MDL, versus a stochastic global optimization approach as employed by BANJO.

**Table 2:** Functional enrichment analysis for the top 20 hubs

			
GlobalMIT^*^network			
**Hub**	**Degree**	**Enriched function**	**Corrected*****p*****-value**
cce_4432	16	Nitrogen fixation	4.5E-5
cce_3394	16	Nitrogen fixation	1.7E-5
cce_3974	14	Photosynthesis, dark reaction	1.4E-2
cce_0997	13	Photosystem I	1.3E-5
cce_0103	12	Plasma membrane proton-transporting	1.7E-5
cce_0589	11	Signal transducer	9.4E-3
cce_1620	10	Photosystem II reaction center	2E-2
cce_1578	10	Structural constituent of ribosome	1E-2
cce_2038	10	Response to chemical stimulus	4.5E-2
cce_4486	9	Photosynthetic membrane	3.1E-2
***BNFinder+MDL network***			
cce_3394	20	Nitrogen fixation	3.7E-8
cce_3377	17	Proton-transporting ATPase activity	2.1E-7
cce_3898	15	Structural constituent of ribosome	2.5E-11
cce_1943	11	peptidoglycan biosynthetic process	3.4E-2
cce_2639	9	thiamine-phosphate kinase activity	2.1E-2
cce_1620	8	Photosystem II reaction center	1E-2
***BANJO network***			
cce_4663	10	Calcium ion binding	3.4E-2

## Conclusion

In this paper, we have introduced GlobalMIT^+^ and GlobalMIT^*^, two DBN-based algorithms for reconstructing gene regulatory networks. The GlobalMIT suite makes use of the recently introduced MIT scoring metric, which is built upon solid principles of information theory, having competitive performance compared against the other traditional scoring metrics such as BIC/MDL and BDe. In this work, we have further shown that MIT possesses another very useful characteristic in that when placed into a deterministic global optimization framework, its complexity is very reasonable. As theoretically shown and experimentally verified, GlobalMIT exhibits a much lower complexity compared to the BDe-based algorithm, i.e., BNFinder+BDe, and is comparable with the MDL-based algorithm, i.e., BNFinder+MDL. GlobalMIT^+/*^ are also designed to learn high-order variable time delayed genetic interactions that are common to biological systems. Furthermore, the GlobalMIT^*^ variant has the capability of reconstructing relatively large-scale networks. As shown in our experiments, GlobalMIT^+/*^ are able to reconstruct genetic networks with biologically plausible structure and enriched submodules significantly better than the alternative DBN-based approaches. Our current and future study of GlobalMIT^+/*^ mainly focuses on the application of these newly developed algorithms to elucidate the gene regulatory network of *Cyanothece*, *Synechocystis*, *Synechococcus* amongst other cyanobacteria strains having high potential for biofuel production and carbon sequestration.

## Competing interests

The authors declare that they have no competing interests.

## Authors’ contributions

NXV developed the algorithms and carried out the experiments. MC provided overall supervision and leadership to the research. NXV and MC drafted the manuscript. RC and PPW suggested the biological data and provided biological insights. All authors read and approved the final manuscript.

## Supplementary Material

Additional file 1 GlobalMIT+.zip — The GlobalMIT^+^ toolbox Implementation of the proposed algorithms in Matlab and C++, together with the user’s guide [15,18-21,23-27,29-31,45-50]. Click here for file

Additional file 2 Supplementary Material for Gene Regulatory Network Modeling via Global Optimization of High-Order Dynamic Bayesian Network [15,34,51]. Click here for file
