# Supplementary material for: Gene regulatory network modeling via global optimization of high-order dynamic Bayesian network
Source: BMC Bioinformatics. 2012 Jun 13;13:131. doi: 10.1186/1471-2105-13-131 (PMC3433362; doi:10.1186/1471-2105-13-131)
Supplement: Additional file 1 — GlobalMIT+.zip — The GlobalMIT+ toolbox Implementation of the proposed algorithms in Matlab and C++, together with the user’s guide [15,18-21,23-27,29-31,45-50]. [file 1471-2105-13-131-S1.zip › GlobalMIT_2.Beta/Doc/globalMIT+_userGuide.pdf]

The GlobalMIT^{+} Toolkit

for

Learning Globally Optimal
High-Order Dynamic Bayesian Network

User Guide

Rev. 2.0
Maintained by Nguyen Xuan Vinh

MONASH University

© 2010-2011 Nguyen Xuan Vinh
All rights reserved

Monash University, Victoria, Australia.

Project members:

- Nguyen Xuan Vinh, Gippsland School of Information Technology, Monash University, Victoria, Australia.
- Madhu Chetty, Gippsland School of Information Technology, Monash University, Victoria, Australia.
- Ross Coppel, Faculty of Medicine, Nursing and Health Sciences, Monash University, Victoria, Australia.
- Pramod P. Wangikar, Chemical Engineering Department, Indian Institute of Technology Bombay, India.

First edition: 10 Feb 2011

2nd edition: 8 Jun 2011

3rd edition: 14 Sept 2011

Contact: {vinh.nguyen,madhu.chetty}@monash.edu

Project website: http://code.google.com/p/globalmit/

Short contents

Short contents · iii

Contents · iv

1 User Manual · 1

2 Supplementary Material · 11

Bibliography · 19

Contents

Short contents iii

Contents iv

1 User Manual 1
1.1 Introduction 1
1.2 Installation 1
1.3 Usage and Examples 2
1.3.1 First-order DBN 2, *1.3.2 High-order DBN* 5, *1.3.3 Analyzing large data set with GlobalMIT** 6, *1.3.4 Using GlobalMIT$^{+/*}$ with prior Transcription Factor information* 8, *1.3.5 Using GlobalMIT^{+} without Matlab* 9, *1.3.6 Efficient Implementation for GlobalMIT^{+}* 9

2 Supplementary Material 11
2.1 The MIT scoring metric for learning DBN 11
2.2 The GlobalMIT^{+} algorithm 13
2.2.1 Complexity analysis 16, *2.2.2 The GlobalMIT* algorithm* 17

Bibliography 19

One

User Manual

1.1 Introduction

GlobalMIT$^+$ is a Matlab/C++ toolkit for learning high-order dynamic Bayesian network (DBN) structure. It implements our polynomial time algorithm for learning the globally optimal high-order DBN structure using the Mutual Information Test (MIT) criterion, as presented in [VCCWon]. The DBN model assumed by GlobalMIT$^+$ is the dth-order Markov stationary DBN, in which both the structure of the network and the parameters characterizing it are assumed to remain unchanged over time.

Generally, DBN models consist of two parts: the *prior network* and the *transition network* [FMR98]. The prior network contains only intra time slice edges (since there are no other time slices preceding it), while the transition network can contain both inter and intra time slice edges, as demonstrated in Fig. 1.1(a,b). Learning the prior network requires collecting m independent observation sequences, of which only m initial time slices are used for learning. For biological networks, such data abundance is not always available, since there may be only one or a very limited number of time series. Therefore, only the learning of the transition network is practical and is relevant. *Henceforth, by DBN we mean only the transition network part of the model*. Some authors have further restricted the transition network to contain only inter time slice edges [Hus03, Doj06, WD09]. *GlobalMIT$^+$ also learn DBN with only inter-time slice edges*, as exemplified in Fig. 1.1(c).

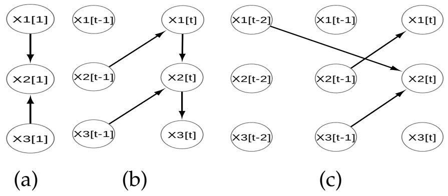

Figure 1.1: (a) prior network; (b) First-order Markov transition network; (c) 2nd-order Markov transition network with only inter time slice edges.

1.2 Installation

The GlobalMIT$^+$ Matlab toolbox is ready for use upon adding its container directory to the Matlab path environment variable (File $\rightarrow$ Set path). The main files of the toolbox are listed in Tables 1.1 and 1.2. The Matlab toolbox contains all the functionalities. However, for

improved performance, the C++ search engine can also be used. We provide pre-compiled executable version of this search engine for Windows and Linux Debian 2.6.32-35. In other environments, the users may recompile the search engine. For example, in Linux, one may use:

```
g++ -g globalMIT.cpp -o globalMIT.exe
```

Table 1.1: GlobalMIT Matlab toolbox main functions

File	Description
globalMIT.m	The Matlab GlobalMIT$^+$ DBN search engine
globalMIT_fixedOrder.m	The Matlab GlobalMIT* DBN search engine (fixed-order version)
globalMIT_exe.m	Matlab interface for the GlobalMIT$^+$ C++ search engine
globalMIT_fixedOrder_exe.m	Matlab interface for the GlobalMIT* C++ search engine (fixed-order data)
myDataMapping.m	Map discrete data to a continuous value range
myIntervalDiscretize.m	Interval discretize data using equal bins
myQuantileDiscretize.m	Quantile discretize data
compare_net.m	Compare a network with a true network
createDotGraphic.m	Create dot graphic (require Graphviz)
create_SIF_file.m	Create SIF graphic file
demo.m	A walk-through demonstration

Table 1.2: GlobalMIT$^+$ C++ search engines

File	Description
globalMIT.cpp	the GlobalMIT$^+$ DBN search engine
globalMIT_fixedOrder.cpp	the GlobalMIT* DBN search engine

GlobalMIT$^+$ can handle data that is a single time series, or a concatenation of multiple time series. In the latter case, the information on the length or each time series in the correct order must be supplied to GlobalMIT$^+$, so that the data are correctly aligned, as in Fig. 1.2.

1.3 Usage and Examples

In this section, we demonstrate the use of GlobalMIT$^+$ for DBN structure learning via a set of walk-through examples. The code for these examples can be found in the `demo.m` file.

1.3.1 First-order DBN

We first load the yeast data set [Hus03]:

```
load husmeier_yeast_100;
```

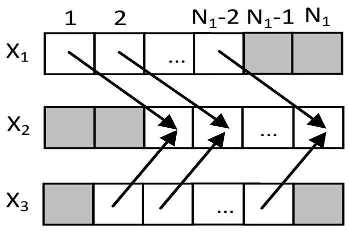

Figure 1.2: Multi time series data alignment for the DBN in Fig. 1.1c.

The data file contains a true network, and synthetic data generated from this network as described in [Hus03]. The synthetic data set is a single time series of 100 discrete observations. The data were generated using Husmeier's program[1], assuming a 1st-order DBN model. We first visualize this network using Graphviz, an open source graph visualization software from AT&T Research[2]. The resulting graph is displayed in Fig. 1.3.

```
createDotGraphic(true_net,nodeName,true_net,'True DBN');
```

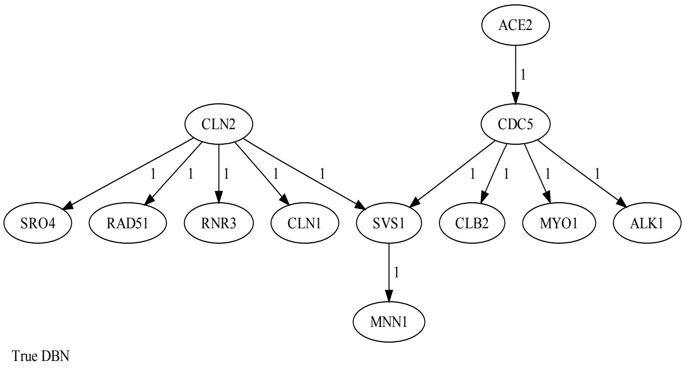

Figure 1.3: True yeast network.

This data set is binary, so we do not need to discretize the data. We use the Matlab GlobalMIT$^+$ search engine to find the optimal DBN:

```
alpha=0.999;
allowSelfLoop=0;
```

[1]http://www.bioss.ac.uk/∼dirk/msc/Software/SyntheticTimeSeriesFriedYeast.m

[2]Graphviz should be downloaded and installed from www.graphviz.org. The Graphviz execution folder should be on the system path.

```
order=1;
dataLength=[100];
[best_net]=globalMIT(data,alpha,order,allowSelfLoop,dataLength);
createDotGraphic(best_net,nodeName,true_net,'GlobalMIT DBN');
```

This function takes the following parameters:

- `data`: a single or multiple time series data, with each column being a variable, and each row being an observation.
- `alpha`: the significance level for the mutual information test of independence.
- `order`: order of the DBN
- `allowSelfLoop`: allow the self regulated link (a link from a node to it-self) or not
- `dataLength`: a vector containing the length of the time series in respective order. For example, if the data matrix is of size 100×20, corresponding to 20 nodes and 100 time points consisting of 3 separate time series of length 20, 30, 50 concatenated in that order then `dataLength=[20,30,50];`

The output of this algorithm is an adjacency matrix, with entries taking values from 0 to `order`, corresponding to the order of regulation. GlobalMIT$^+$ updates information as it goes through the nodes, and output the best parents leant for each node, along with the order information. It can be seen in Figure 1.4 that GlobalMIT$^+$ finds the correct DBN as in Fig. 1.3.

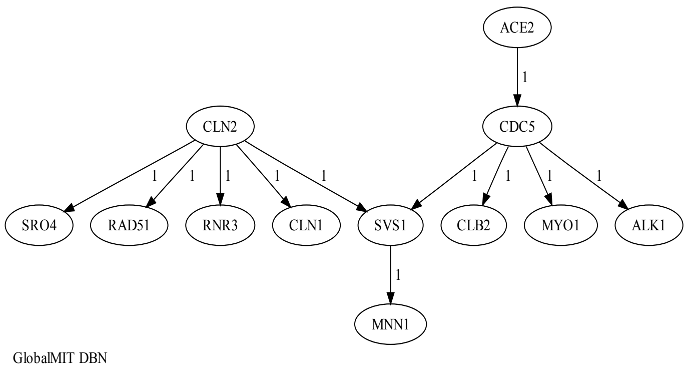

Figure 1.4: DBN found by GlobalMIT$^+$.

On our Quad-core i7 PC with 8Gb of main memorry, this operation takes:

```
Elapsed time is 15.672523 seconds.
```

We now test the C++ version of GlobalMIT$^+$:

```
nthreads=1;
best_net_exe=globalMIT_exe(data,alpha,order,nthreads,allowSelfLoop,dataLength);
createDotGraphic(best_net_exe,nodeName,true_net,'GlobalMIT C++ DBN');
```

This function takes an additional parameter:

- `nthreads`: the number of computation threads

It can be verified that GlobalMIT$^+$ C++ finds the network identical to GlobalMIT$^+$ Matlab. On the same computer, GlobalMIT$^+$ C++ takes only a fraction of a second to complete the task:

```
Elapsed time is 2.355533 seconds.
```

On multicore system, the number of threads can be increased to improve the performance further.

Now we test GlobalMIT$^+$ with multi time series data. Pretending that the data is made up of 3 time series of 20, 30 and 50 observations, then:

```
dataLength=[20,30,50];
best_net_exe=globalMIT_exe(data,alpha,order,nthreads,allowSelfLoop,dataLength);
```

Note how GlobalMIT$^+$ reports this change:

```
N= 100 Dim= 12
Number of time series= 3
Number of effective samples= 97
```

1.3.2 High-order DBN

On the same yeast network data set, we increase the order of the DBN to 3:

```
dataLength=[100];
order=3;
best_net_exe=globalMIT_exe(data,alpha,order,nthreads,allowSelfLoop,dataLength);
createDotGraphic(best_net,nodeName,true_net,'GlobalMIT DBN');
```

GlobalMIT$^+$ requires significantly more time to complete this task (258 secs). It returns the same network as in Fig 1.4. Our Quad-core i7 PC can handle 8 threads concurrently. We thus increase the number of threads to 6 and repeat the experiment:

```
nthreads=6;
best_net_exe=globalMIT_exe(data,alpha,order,nthreads,allowSelfLoop,dataLength);
```

The Matlab interface then calls 6 C++ engine threads concurrently. The runtime reduces to just 52 secs.

It is noted that this data set contains only 1st-order interactions by the way the data were created. We now introduce some "artificial" higher-order time-delayed interactions into the network by shifting some gene expression profiles ahead in time as follows:

```
load husmeier_yeast_100;
for i=1:length(nodeName) eval(sprintf('%s=%d;',nodeName{i},i));end;
data(:,[RAD51  RNR3])=vecShift(data(:,[RAD51  RNR3]),1);
data(:,[CLB2 MYO1 ALK1])=vecShift(data(:,[CLB2 MYO1 ALK1]),2);
```

By doing this, the interactions between CLN2 and {RAD51, RNR3} become 2nd-order interactions, and those between CDC5 and {CLB2, MYO1, ALK1} become 3rd-order interactions.

```
order=3;
[best_net_exe]=globalMIT_exe(data,alpha,order,nthreads,allowSelfLoop,dataLength);
createDotGraphic(best_net_exe,nodeName,true_net,'GlobalMIT DBN');
```

GlobalMIT$^+$ with order 3 reconstruct the correct network with correct order, as in Figure 1.5.

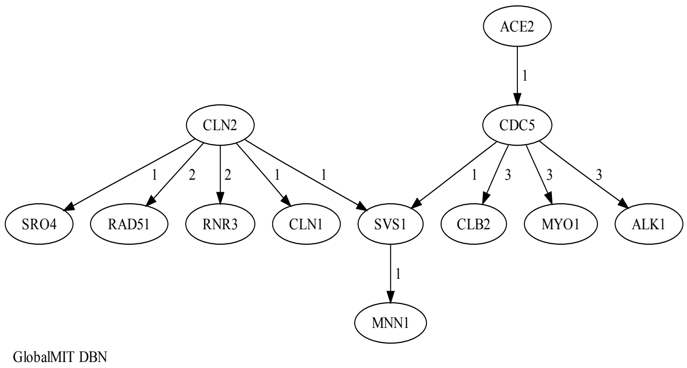

Figure 1.5: High-order DBN found by GlobalMIT$^+$.

1.3.3 Analyzing large data set with GlobalMIT*

This section illustrates the use of GlobalMIT* on large data sets. The worst case complexity of GlobalMIT$^+$ is dependant upon the number of variables, the number of observations and the DBN order. Since the analysis of large data set using higher-order DBN may be very time consuming, the GlobalMIT* variant requires that for every pair of nodes, there can be only a single link at a pre-determined time lag where their mutual information is maximized. As such, the time complexity of GlobalMIT* is always equivalent to that of the first-order GlobalMIT.

We first load the following data set:

```
clear clc;
load Yu_net_5;
n_state=3;
```

which is generated from Yu's net No. 1 [YSW$^+$04], as demonstrated in Fig. 1.6. This network consists of 20 nodes, and operates according to the following linear dynamical system:

$$X_{t+1} - X_t = A(X_t - T) + \epsilon \quad (1.1)$$

with X denotes the expression profiles, A describes the strength of gene-gene regulations, T is the constitutive expression values, and ϵ simulates a uniform biological noise. The detailed parameters can be found in [YSW+04].

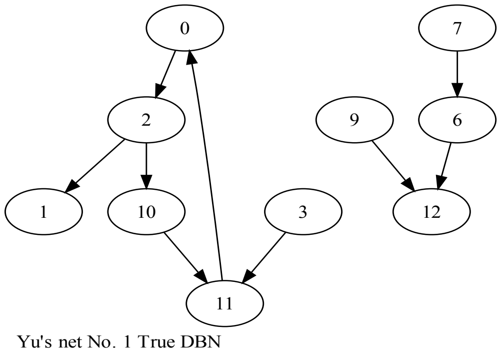

Figure 1.6: Yu's net 1.

The data contains 20 nodes×300 observations. We discretize the data using 3-state quantile discretization, then run GlobalMIT$^+$ with network order set to 5.

```
alpha=0.9999;
allowSelfLoop=1;
order=5;
nthreads=6;
data_dis= myIntervalDiscretize(data,n_state);
[dataLength dim]=size(data_dis);
[best_net_exe]=globalMIT_exe(data_dis,alpha,order,nthreads,allowSelfLoop,dataLength);
createDotGraphic(best_net_exe,[],true_net,'GlobalMIT+ C++ DBN');
compare_net(best_net_exe,true_net,1)
```

The C++ implementation of GlobalMIT$^+$ would take slightly more than an hour on our core i7 PC (with 6 threads running in parallel) to analyze this data set. The reconstructed network is presented in Fig. 1.7. The quality metrics for this reconstructed network, namely precision (PPV) and sensitivity, are:

```
PPV= 1.000000  Sensitivity: 0.666667
```

We now check the GlobalMIT* version:

```
[best_net_exe]=globalMIT_exe(data_dis,alpha,order,nthreads,allowSelfLoop,dataLength);
createDotGraphic(best_net_exe,[],true_net,'GlobalMIT+ C++ DBN');
compare_net(best_net_exe,true_net,1)
```

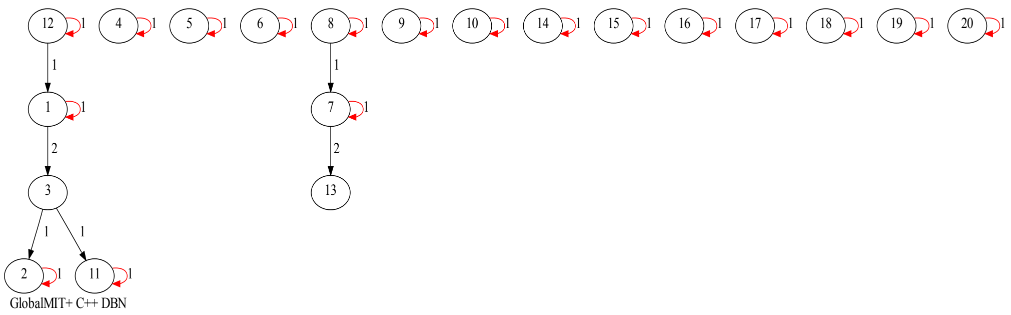

Figure 1.7: GlobalMIT$^+$ reconstruction result for the Yu's net No 1

The GlobalMIT* version takes only less than a minute, and produces network is presented in Fig. 1.8. The quality metrics for this reconstructed network are:

```
PPV= 1.000000  Sensitivity: 0.555556
```

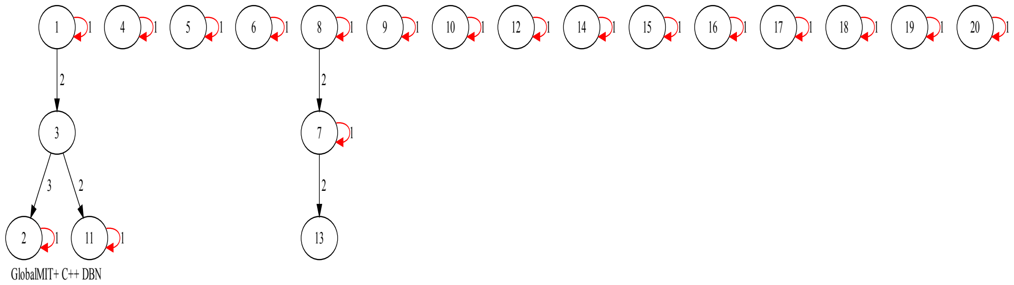

Figure 1.8: GlobalMIT* reconstruction result for the Yu's net No 1

1.3.4 Using GlobalMIT$^{+/*}$ with prior Transcription Factor information

GlobalMIT$^{+/*}$ can take information on which nodes are designated regulatory nodes in the network. In the context of gene regulatory network, these nodes are often transcription factor (TF) genes, that control the transcription rate of other genes. If such information on a list of potential n_{TF} regulatory nodes is known, then the input data should be reorganized in such a way that the first n_{TF} columns of the input matrix correspond to n_{TF} regulatory nodes. Then a variant of the search engine (with postfix "_TF") can be called as follows:

```
[best_net]=globalMIT_fixedOrder_TF(a,alpha,d,allowSelfLoop,dataLength,nTF)
```

where `n_TF` is the number of regulatory nodes. GlobalMIT$^{+/*}$ will learn an optimal network in which only regulatory nodes are allowed to have out-going links.

1.3.5 Using GlobalMIT^{+} without Matlab

It is noted that GlobalMIT^{+} does not require Matlab, a commercial software, to carry out its core functionality, i.e., searching for the globally optimal DBN under MIT, thanks to the GlobalMIT^{+} C++ implementation. Note however that GlobalMIT^{+} C++ requires access to the inverse Chi square function. This function is available in the GNU scientific library. However, to keep maximal portability, the current version of GlobalMIT^{+} C++ requires this information to be pre-provided in a file (`myChiValue.txt`, currently created by the `writeGlobalMITfile.m` interface module). However, the user can use Octave, a freely available, Matlab-like software, which also provides the inverse Chi square function, to generate this file.

1.3.6 Efficient Implementation for GlobalMIT^{+}

The search procedure involves examining all potential parent sets of increasing cardinality. The following decomposition property of the mutual information is handy when it comes to design an efficient implementation for GlobalMIT^{+} :

$$I(X_i, \mathbf{Pa}_i \cup X_j) = I(X_i, \mathbf{Pa}_i) + I(X_i, X_j|\mathbf{Pa}_i)$$

This implies that the mutual information (MI) can be computed incrementally, and suggests that, for efficiency, the computed mutual information values should be cached to avoid redundant computations, subject to memory availability. During execution, you may see GlobalMIT^{+} reports the amount of memory currently being used for MI caching:

```
Node 20 Pstar= 5
P=1 Allocating 160 bytes = 0.000152588 Mb of MI Cache.
P=2 Allocating 1520 bytes = 0.00144958 Mb of MI Cache.
P=3 Allocating 9120 bytes = 0.00869751 Mb of MI Cache.
P=4 Allocating 38760 bytes = 0.0369644 Mb of MI Cache.
```

Two

Supplementary Material

In this section, we review the MIT score for learning BN, then adapts it to the high order DBN case. The GlobalMIT$^{+/*}$ are presented afterwards. For a more complete treatment of the subject, interested readers are referred to [VCCWon].

2.1 The MIT scoring metric for learning DBN

In this section, we first review the MIT score for learning BN and then show how it can be adapted to the DBN case. The most popular approaches for learning DBN are essentially those that have been adapted from the static BN literature, namely the *search+score* paradigm [YSW+04, WD09], and Markov Chain Monte Carlo (MCMC) simulation [Hus03, DLH10, RH10]. In this paper we apply the *search+score* approach, in which we specify a scoring function to assess the goodness-of-fit of a DBN given the data, and a search procedure to find the optimal network based on this scoring metric. Several popular scores for static BN, such as the Bayesian scores (K2, BD, BDe and BDeu), and the information theoretic scores (BIC/MDL, Akaike Information Critetion—AIC), can be adapted directly for DBNs.

Mutual Information Test (MIT) is a recently introduced scoring metric for learning BN [dC06]. Briefly speaking, under MIT the goodness-of-fit of a network is measured by the total mutual information shared between each node and its parents, penalized by a term which quantifies the degree of statistical significance of this shared information. To understand MIT, let $\{r_1, \ldots, r_n\}$ be the number of discrete states corresponding to our set of RVs $\mathbf{X} = \{X_1, \ldots, X_n\}$, D denote our data set of N observations, G be a BN, and $\mathbf{Pa}_i = \{X_{i1}, \ldots, X_{is_i}\}$ be the set of parents of X_i in G with corresponding $\{r_{i1}, \ldots, r_{is_i}\}$ discrete states, $s_i = |\mathbf{Pa}_i|$, then the MIT score is defined as:

$$S_{MIT}(G : D) = \sum_{i=1;\mathbf{Pa}_i \neq \emptyset}^{n} \{2N \cdot I(X_i, \mathbf{Pa}_i) - \sum_{j=1}^{s_i} \chi_{\alpha, l_{i\sigma_i(j)}}\},$$

where $I(X_i, \mathbf{Pa}_i)$ is the mutual information between X_i and its parents as estimated from D. $\chi_{\alpha,l_{ij}}$ is the value such that $p(\chi^2(l_{ij}) \leq \chi_{\alpha,l_{ij}}) = \alpha$ (the Chi-square distribution at significance level $1-\alpha$), and the term $l_{i\sigma_i(j)}$ is defined as:

$$l_{i\sigma_i(j)} = \begin{cases} (r_i - 1)(r_{i\sigma_i(j)} - 1) \prod_{k=1}^{j-1} r_{i\sigma_i(k)}, & j = 2 \ldots, s_i \\ (r_i - 1)(r_{i\sigma_i(j)} - 1), & j = 1 \end{cases}$$

where $\sigma_i = \{\sigma_i(1), \ldots, \sigma_i(s_i)\}$ is any permutation of the index set $\{1 \ldots s_i\}$ of $\mathbf{Pa}_i$, with the first variable having the greatest number of states, the second variable having the second largest number of states, and so on.

To make sense of this criterion, let us first point out that maximizing the first term in the score, $\sum_i 2N \cdot I(X_i, \mathbf{Pa}_i)$, can be shown to be equivalent to maximizing the log-likelihood criterion. However, learning BN by using the maximum likelihood principle suffers from overfitting, as the fully-connected network will always have the maximum likelihood. Likewise, for the MIT criterion, since the mutual information can always be increased by including additional variables to the parent set, i.e., $I(X_i, \mathbf{Pa}_i \cup X_j) \geq I(X_i, \mathbf{Pa}_i)$, the complete network will have the maximum total mutual information. Thus, there is a need to penalize the complexity of the learned network. Penalizing the log-likelihood criterion with $-\frac{1}{2}C(G)\log(N)$ gives us the BIC/MDL criteria, while $-C(G)$ gives us the AIC criterion (where $C(G) = \sum_{i=1}^{n}(r_i - 1)\prod_{j=1}^{s_i} r_{ij}$ measures the network complexity). As for the MIT criterion, while the mutual information always increases when including additional variables to the parent set, the degree of statistical significance of this increment might become negligible as more and more variables are added. This significance degree can be quantified based on a classical result in information theory by [Kul68], which, in this context, can be stated as follows: under the hypothesis that X_i and X_j are conditionally independent given $\mathbf{Pa}_i$ is true, the statistics $2N \cdot I(X_i, X_j|\mathbf{Pa}_i)$ approximates to a $\chi^2(l)$ distribution, with $l = (r_i - 1)(r_j - 1)q_i$ degree of freedom, and $q_i = 1$ if $\mathbf{Pa}_i = \emptyset$, otherwise q_i is total the number of state of $\mathbf{Pa}_i$, i.e., $q_i = \prod_{k=1}^{s_i} r_{ik}$. Thus the second term in the MIT score penalizes the addition of more variables to the parent set. Roughly speaking, only variables that have the conditional mutual information shared with X_i given all the other variables in $\mathbf{Pa}_i$ that is higher than 100α percent of the MI values under the null hypothesis of independence can increase the score. An important difference between MIT and the other information theoretic based metrics (BIC/MDL, AIC) is that the penalty term is applied individually and independently to each RV rather than to the network as a whole. For further details on the motivation and derivation of this scoring metric as well as an extensive comparison with BIC/MDL and BD, we refer readers to [dC06].

We next show how MIT can be adapted for the case of high-order DBN learning. This is rather straightforward, provided we pay due attention to data alignment: the mutual information is now calculated between a parent set and its child, possibly at various time lags. At any time $t > d$, let $\mathbf{Pa}_i = \{X_{i1}[t - \delta_{i1}], \ldots, X_{is_i}[t - \delta_{is_i}]\}$ be the parent set of $X_i[t]$, with $\{\delta_{i1}, \ldots, \delta_{is_i}\}$ be the actual regulation order corresponding to each parent. In this work, since we only consider DBN with inter time slice edges, $1 \leq \delta_{ij} \leq d, \forall j$ for a d-th order DBN. When the mutual information is calculated, the target node should always be shifted d units forward in time, while the parents should be shifted $\{d - \delta_{i1}, \ldots, d - \delta_{is_i}\}$ time units forward respectively. We define I_s as a special mutual information operator, which automatically shifts the target variable as well as all of its parents to the correct alignment. The number of *effective observations* N_e is therefore $N_e = N - d$, if we have only one observation sequence of length N. In cases where we have m separate time series, it is imperative that there are no wrong alignments at the transition between these time series when they are concatenated. The number of effective observations for multiple time series is $N_e = \sum_{i=1}^{m} N_i - md$. The MIT score for DBN should be calculated as:

$$S'_{MIT}(G : D) = \sum_{i=1;\mathbf{Pa}_i \neq \emptyset}^{n} \{2N_e \cdot I_s(X_i, \mathbf{Pa}_i) - \sum_{j=1}^{s_i} \chi_{\alpha, l_{i\sigma_i(j)}}\}.$$

To make this clear, we demonstrate the process of data alignment through the simple DBN example given in Fig. 1.1(c). For node X_2, $\mathbf{Pa}_2 = \{X_1[t-2], X_3[t-1]\}$, therefore when $I_s(\cdot)$ operates, it shifts the target node X_2 forward by two units in time, while the parent X_1 is shifted zero unit, and parent X_3 is shifted 1 unit, as shown in Fig. 2.1. The number of effective observations is $N_e = N_1 - 2$ if only the first sequence is used, or $N_e = N_1 + N_2 + N_3 - 2 \times 3$ if all 3 sequences are used for learning.

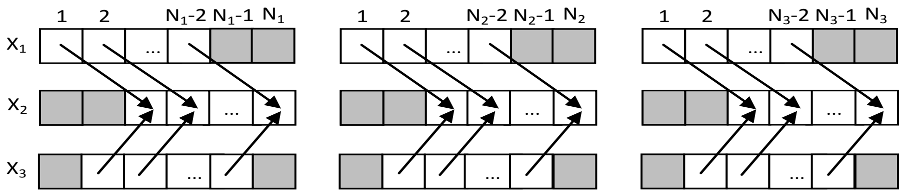

Figure 2.1: Data alignment for node X_2 in the DBN in Fig. 1.1(c). Shaded cells denote unused observations for the calculation of $I_s(X_2, \mathbf{Pa}_2)$.

2.2 THE GLOBALMIT$^+$ ALGORITHM

This section presents our GlobalMIT$^+$ algorithm for learning the globally optimal structure for a d-th order DBN with the MIT scoring metric in polynomial time. The original GlobalMIT algorithm for the case of the 1st-order Markov DBN [VCCW11] can be obtained as a special case of GlobalMIT$^+$ when $d = 1$. Our development made use of the same set of assumptions proposed by [Doj06]. While Dojer considered the DBN learning problem in a generic machine learning context, herein we shall focus our attention to the particular context of GRN modeling. Next, we list the required assumptions and discuss their associated rationales and biological plausibility.

Assumption 1. *(acyclicity) Examination of the graph acyclicity is not necessary.*

This assumption is valid for DBNs with no inter time slice edges. For this class of DBN, as the edges are directed only forward in time, acyclicity is automatically satisfied. The biological implication of this assumption is that we may not be able to detect the instantaneous interactions. As discussed previously, genetic interactions are generally time-delayed. Only when the sampling gap is large, we may consider some quick interactions as instantaneous. The effect of this restriction is that, if gene X_1 regulates gene X_2 almost instantly, their mutual information $I(X_1, X_2)$ will likely be maximized when their expression profiles are in synchrony, i.e., no shifting of any of the two sequences is involved. With Assumption 1 in place, we will have to consider two mutual information values, $I_s(X_1, X_2)$ and $I_s(X_2, X_1)$, since I_s is asymmetric. If these values are significantly weaker than $I(X_1, X_2)$ then the interaction between genes X_1 and X_2 may be undetected. However, when the signal is smooth and is sampled densely enough, we have observed that shifting the expression profile just one time unit will not often cause a large reduction in the MI value. This is because smooth time series have high auto-correlation at short lags, and thus, instantaneous interactions may still be captured by DBN models with only inter-time slice edges. The algorithmic implication of assumption 1 becomes clear when we consider assumption 2 below:

Assumption 2. *(additivity)* $S(G : D) = \sum_{i=1}^{n} s(X_i, \boldsymbol{Pa}_i : D|_{X_i \cup \boldsymbol{Pa}_i})$ *where* $D|_{X_i \cup \boldsymbol{Pa}_i}$ *denotes the restriction of* D *to the values of the members of* $X_i \cup \boldsymbol{Pa}_i$.

To simplify notation, we write $s(\mathbf{Pa}_i)$ for $s(X_i, \mathbf{Pa}_i : D|_{X_i \cup \mathbf{Pa}_i})$. Assumption 2 simply states that the scoring function decomposes over the variables, which is satisfied by most scoring metrics such as BIC/MDL, BD and also clearly by MIT. However together with assumption 1, their algorithmic implication is profound: these assumptions allow us to compute the parents set of each node independently. Unlike the case of BN where the choice of parents for a certain node may affect the choice of parents of all the other nodes, for DBN (without intra time slice edges), the choice of parents for a node has no effect on the other nodes. Thus the algorithms developed based upon these two assumptions become very amenable to parallelization, i.e., each node can be learned independently with a separate computational thread. Still, exhaustive brute-force search for the optimal parent set requires exponential time, as for a d-th order DBN, $\mathbf{Pa}_i$ can be an arbitrary subset of $\mathbf{X}[t-1] \cup \ldots \cup \mathbf{X}[t-d]$, and the number of all possible parent sets is 2^{dn}. In order to further reduce the search space, we need to rely on the special structure of the scoring metric, as follows:

Assumption 3. *(splitting)* $s(\boldsymbol{Pa}_i) = u(\boldsymbol{Pa}_i) + v(\boldsymbol{Pa}_i)$ *for some non-negative functions* u *and* v *satisfying* $\boldsymbol{Pa}_i \subseteq \boldsymbol{Pa}'_i \Rightarrow u(\boldsymbol{Pa}_i) \leq u(\boldsymbol{Pa}'_i)$.

Assumption 4. *(uniformity)* $|\boldsymbol{Pa}_i| = |\boldsymbol{Pa}'_i| \Rightarrow u(\boldsymbol{Pa}_i) = u(\boldsymbol{Pa}'_i)$.

Assumption 3 requires the scoring function to decompose into two components: v evaluating the accuracy of representing the distribution underlying the data by the network, and u measuring its complexity. Furthermore, u is required to be a monotonically non-decreasing function in the cardinality of $\mathbf{Pa}_i$ (Assumption 4), i.e., the network gets more complex as more variables are added to the parent sets. However in its original form, the MIT scoring metric, having higher scores for better networks, does not abide by these assumptions. We can overcome this by casting the problem as a minimization problem (similar to Dojer) where lower scored networks are better. We consider a variant of MIT as follows:

$$S_{MIT}(G : D) \quad = \quad \sum_{i=1}^{n} 2N_e \cdot I_s(X_i, \mathbf{X}^d) - S'_{MIT}(G : D), \tag{2.1}$$

where $\mathbf{X}^d = \mathbf{X}[t-1] \cup \ldots \cup \mathbf{X}[t-d]$. This score admits the following decomposition over each variable (with the convention of $I(X_i, \emptyset) = 0$):

$$s_{MIT}(\mathbf{Pa}_i) \quad = \quad v_{MIT}(\mathbf{Pa}_i) + u_{MIT}(\mathbf{Pa}_i), \tag{2.2}$$

$$v_{MIT}(\mathbf{Pa}_i) \quad = \quad 2N_e \cdot I_s(X_i, \mathbf{X}^d) - 2N_e \cdot I_s(X_i, \mathbf{Pa}_i), \tag{2.3}$$

$$u_{MIT}(\mathbf{Pa}_i) \quad = \quad \sum_{j=1}^{s_i} \chi_{\alpha, l_{i\sigma_i(j)}}. \tag{2.4}$$

Roughly speaking, v_{MIT} measures the "error" of representing the joint distribution underlying D by G, while u_{MIT} measures the complexity of this representation. We state the following results:

Proposition 1. S'_{MIT} *maximization is equivalent to* S_{MIT} *minimization.*

Proof. This is obvious, since $\sum_{i=1}^{n} 2N_e \cdot I_s(X_i, \mathbf{X}^d) = constant$. □

Proposition 2. *v_{MIT}, u_{MIT} satisfy assumption 3.*

Proof. $v_{MIT} \geq 0$ since of all possible parent sets $\mathbf{Pa}_i$, the full set $\mathbf{X}^d$ has the maximum (shifted) mutual information with X_i. And since the support of the Chi-square distribution is $\mathbb{R}^+$, i.e., $\chi_{\alpha,\cdot} \geq 0$, therefore $\mathbf{Pa}_i \subseteq \mathbf{Pa}'_i \Rightarrow 0 \leq u_{MIT}(\mathbf{Pa}_i) \leq u_{MIT}(\mathbf{Pa}'_i)$. □

While we note that u_{MIT} does not satisfy Assumption 4, for applications where all the variables have the same number of states, it can be shown to satisfy this assumption. Within the context of GRN modeling from microarray data, this generally holds true, since it is a popular practice to discretize expression data of all genes to, e.g., 3 states corresponding to the gene being up, down and base-line regulated [YSW+04].

Assumption 5. *(variable uniformity) All variables in $\mathbf{X}$ have the same number of discrete states k.*

Proposition 3. *Under the assumption of variable uniformity, u_{MIT} satisfies assumption 4.*

Proof. It can be seen that if $|\mathbf{Pa}_i| = |\mathbf{Pa}'_i| = s_i$, then $u_{MIT}(\mathbf{Pa}_i) = u_{MIT}(\mathbf{Pa}'_i) = \sum_{j=1}^{s_i} \chi_{\alpha,(k-1)^2 k^{j-1}}$. □

Since $u_{MIT}(\mathbf{Pa}_i)$ is the same for all parent sets of the same cardinality, we can write $u_{MIT}(|\mathbf{Pa}_i|)$ in place of $u_{MIT}(\mathbf{Pa}_i)$. With Assumptions 1-5 satisfied, we can employ the following Algorithm 1, named globalMIT$^+$, to find the globally optimal DBN with MIT, i.e., the one with the minimal S_{MIT} score.

Algorithm 1 GlobalMIT$^+$: Optimal d^{th}-order DBN with MIT

```
Pa_i := ∅
for p = 1 to nd do
   If u_MIT(p) ≥ s_MIT(Pa_i) then return Pa_i; Stop.
   P = arg min{s_MIT(Y)|Y ⊆ X^d; |Y| = p}
   If s_MIT(P) < s_MIT(Pa_i) then Pa_i := P.
end for
```

Theorem 1. *Under assumptions 1-5, GlobalMIT$^+$ applied to each variable in $\mathbf{X}$ finds a globally optimal d-th order DBN under the MIT score.*

Proof. The key point here is that once a parent set grows to a certain extent, its complexity alone surpasses the total score of a previously found sub-optimal parent set. In fact, all the remaining potential parent sets $\mathbf{P}$ omitted by the algorithm have a total score higher than the current best score, i.e., $s_{MIT}(\mathbf{P}) \geq u_{MIT}(|\mathbf{P}|) \geq s_{MIT}(\mathbf{Pa}_i)$, where $\mathbf{Pa}_i$ is the last sub-optimal parent set found. □

We note that the terms $2N_e \cdot I_s(X_i, \mathbf{X}^d)$ in the S_{MIT} score in (2.1) are all constant and would not affect the outcome of our optimization problem. Knowing their exact value is however, necessary for the stopping criterion in Algorithm 1, and also for constructing its complexity bound, as will be shown in Sec. 2.2.1. Calculating $I_s(X_i, \mathbf{X}^d)$ is by itself a

hard problem, requiring $O(k^{nd+1})$ space and time in general. However, for our purpose, since the only requirement for v_{MIT} is that it must be non-negative, it is sufficient to use an upper bound of $I_s(X_i, \mathbf{X}^d)$. A fundamental property of the mutual information states that $I(\mathbf{U}, \mathbf{V}) \leq \min\{H(\mathbf{U}), H(\mathbf{V})\}$, i.e., mutual information is bounded by the corresponding entropies. We therefore have:

$$2N_e \cdot I_s(X_i, \mathbf{X}^d) \leq 2N_e \cdot H_s(X_i),$$

where $H_s(X_i)$ is the entropy of X_i estimated from a d-time-unit shifted expression profile, i.e., $\{x_{i(d+1)}, \ldots, x_{iN}\}$. Otherwise, we can use a universally fixed upper bound for all $H_s(X_i)$, that is $\log k$, then:

$$2N_e \cdot I_s(X_i, \mathbf{X}^d) \leq 2N_e \cdot \log k.$$

Using these bounds, we obtain the following more practical versions of d_{MIT}:

$$v'_{MIT}(\mathbf{Pa}_i) = 2N_e \cdot H_s(X_i) - 2N_e \cdot I_s(X_i, \mathbf{Pa}_i) \quad (2.5)$$

$$v''_{MIT}(\mathbf{Pa}_i) = 2N_e \cdot \log k - 2N_e \cdot I_s(X_i, \mathbf{Pa}_i) \quad (2.6)$$

It is straightforward to show that Algorithm 1 and Theorem 1 are still valid when v'_{MIT} or v''_{MIT} are used in place of v_{MIT}.

2.2.1 Complexity analysis

Theorem 2. *GlobalMIT$^+$ admits a polynomial worst-case time complexity of $O((nd)^{\log_k N_e})$ in the number of variables and DBN order.*

Proof. Our aim is to find a number p^* satisfying $u_{MIT}(p^*) \geq s_{MIT}(\emptyset)$. Clearly, there is no need to examine any parent set of cardinality p^* and over. In the worst case, our algorithm will have to examine all the possible parent sets of cardinality from 1 to $p^* - 1$. We have:

$$u_{MIT}(p^*) \geq s_{MIT}(\emptyset) \Leftrightarrow \sum_{j=1}^{p^*} \chi_{\alpha, l_i \sigma_i(j)} \geq v_{MIT}(\emptyset) = 2N_e \cdot I_s(X_i, \mathbf{X}^d).$$

As discussed above, since calculating v_{MIT} is not convenient, we use v'_{MIT} and v''_{MIT} instead. With v'_{MIT}, p^* can be found as:

$$p^* = \arg\min\{p | \textstyle\sum_{j=1}^{p} \chi_{\alpha, l_i \sigma_i(j)} \geq 2N_e \cdot H_s(X_i)\},$$

while for v''_{MIT}:

$$p^* = \arg\min\{p | \textstyle\sum_{j=1}^{p} \chi_{\alpha, l_i \sigma_i(j)} \geq 2N_e \cdot \log k\}.$$

It can be seen that p^* depends only on α, k and N_e. Since there are $O((nd)^{p^*})$ subsets of $\mathbf{X}^d$ with at most p^* parents, and each set of parents can be scored in polynomial time, GlobalMIT$^+$ admits an overall polynomial worst-case time complexity in the number of variables n and network order d. While p^* does not admit a closed-form solution (since $\chi_{\alpha, l_{ij}}$ cannot be analytically calculated), a large over-estimate of p^* can be provided as follows. Note that $\chi_{\alpha, l_{ij}}$ is the value such that $p(\chi^2(l_{ij}) \leq \chi_{\alpha, l_{ij}}) = \alpha$. Since generally

$\alpha \gg 0.5$, if we take the mean value (corresponding roughly to $\alpha = 0.5$) of the $\chi^2(l_{ij})$ distribution, i.e., l_{ij}, as an under-estimate for $\chi_{\alpha,l_{ij}}$, then:

$$\sum_{j=1}^{p^*} \chi_{\alpha,l_i\sigma_i(j)} \geq 2N_e \cdot \log k \Leftrightarrow \sum_{j=0}^{p^*-1} (k-1)^2 k^j \geq 2N_e \cdot \log k$$
$$\Leftrightarrow (k-1)(k^{p^*} - 1) \geq 2N_e \cdot \log k \Leftrightarrow p^* \geq \log_k(\frac{2N_e \cdot \log k}{k-1} + 1)$$

Assuming $N_e \gg \log k$, we can see that $p^* \sim \log_k(N_e)$, and the algorithm admits an overall complexity of $O((nd)^{\log_k N_e})$. □

Let us now compare this bound with those of the algorithms for learning the globally optimal DBN under the BIC/MDL and BDe scoring metrics as proposed by [Doj06], and implemented in the BNFinder software [WD09]. For BIC/MDL, p^*_{MDL} is given by $\lceil \log_k N_e \rceil$, while for BDe, $p^*_{BDe} = \lceil N_e \log_{\lambda^{-1}} k \rceil$, where the distribution $P(G) \propto \lambda^{\sum |\mathbf{Pa}_i|}$, with a penalty parameter $0 < \lambda < 1$, is used as a prior over the network structures [Doj06]. In general, p^*_{BDe} scales linearly with the number of effective data items N_e, making its value less of practical interest, even for small data sets. On the other hand, it can be seen that the first order GlobalMIT and BNFinder+MDL admits roughly the same worst-case computational complexity.

2.2.2 The GlobalMIT* algorithm

It is noted that the search space has been expanded from $\mathbf{X}[t-1]$ in the case of the 1st-order DBN, to $\mathbf{X}^d = \mathbf{X}[t-1] \cup \ldots \cup \mathbf{X}[t-d]$ for the case of the dth-order DBN. Roughly, the number of variables has been multiplied d times in order to accommodate the higher-order regulations. Such a multiplicative expansion in the search space may be very expensive, especially for a deterministic global optimization algorithm such as GlobalMIT$^+$. For very large networks, it may be useful to consider the following additional assumption:

Assumption 6. *(non-redundant, optimal-lag interaction) There are no multiple edges from a parent X_i to its child X_j at different time lags. Furthermore, the only one edge allowed, if it exists, must take place at the optimal lag δ^*_{ij}, where $\delta^*_{ij} = \arg\max\{I_s(X_j, X_i[t-\delta])|1 \leq \delta \leq d\}$.*

This assumption restricts that for each node X_i there may be only a single link to a node X_j at the optimal time lag where their mutual information is maximized. With this assumption in place, the search space for each variable X_j reduces from $\mathbf{X}^d = \mathbf{X}[t-1] \cup \ldots \cup \mathbf{X}[t-d]$ to $\mathbf{X}^*_j = \{X[t-\delta^*_{ij}]\}_{i=1\ldots n}$, which is equivalent in size to the first-order GlobalMIT algorithm. Thus *assumption 6 provides a trade-off between optimality and coverage*: one one hand, the search is performed only on n variables at a pre-determined lag hence significantly reduces the computational cost. On the other hand, this lag can take any value from 1 to d hence provides a means to detect delayed genetic interactions, at the lag where they are most likely to take place. We shall refer to the variant of GlobalMIT$^+$ when assumption 6 is employed as GlobalMIT*. It is not hard to see that, for any high order $d > 1$, GlobalMIT* still admits the same complexity as the first order GlobalMIT.

This is the end ⌣̈

Bibliography

[dC06] Luis M. de Campos. A scoring function for learning bayesian networks based on mutual information and conditional independence tests. *J. Mach. Learn. Res.*, 7:2149–2187, December 2006.

[DLH10] Frank Dondelinger, Sophie Lebre, and Dirk Husmeier. Heterogeneous continuous dynamic bayesian networks with flexible structure and inter-time segment information sharing. In *ICML*, pages 303–310, 2010.

[Doj06] Norbert Dojer. Learning Bayesian Networks Does Not Have to Be NP-Hard. In *Proceedings of International Symposium on Mathematical Foundations of Computer Science*, pages 305–314, 2006.

[FMR98] Nir Friedman, Kevin Murphy, and Stuart Russell. Learning the structure of dynamic probabilistic networks. In *UAI'98*, pages 139–147. Morgan Kaufmann, 1998.

[Hus03] Dirk Husmeier. Sensitivity and specificity of inferring genetic regulatory interactions from microarray experiments with dynamic Bayesian networks. *Bioinformatics*, 19(17):2271–2282, 2003.

[Kul68] Solomon Kullback. *Information Theory and Statistics*. Dover publications, 1968.

[RH10] Joshua Robinson and Alexander Hartemink. Learning Non-Stationary Dynamic Bayesian Networks. In *the Journal of Machine Learning Research*, volume 11, pages 3647–3680, 2010.

[VCCW11] Nguyen Xuan Vinh, Madhu Chetty, Ross Coppel, and Pramod P. Wangikar. A polynomial time algorithm for learning globally optimal dynamic Bayesian network. In *Proceedings of the 18th International Conference on Neural Information Processing (ICONIP'11)*, 2011.

[VCCWon] Nguyen Xuan Vinh, Madhu Chetty, Ross Coppel, and Pramod P. Wangikar. Gene regulatory network modeling via global optimization of dynamic bayesian network. *Bioinformatics*, 2011-submitted for publication.

[WD09] Bartek Wilczynski and Norbert Dojer. BNFinder: exact and efficient method for learning Bayesian networks. *Bioinformatics*, 25(2):286–287, 2009.

[YSW+04] Jing Yu, V. Anne Smith, Paul P. Wang, Alexander J. Hartemink, and Erich D. Jarvis. Advances to Bayesian network inference for generating causal networks from observational biological data. *Bioinformatics*, 20(18):3594–3603, 2004.
