# Supplementary material for: Gene regulatory network modeling via global optimization of high-order dynamic Bayesian network
Source: BMC Bioinformatics. 2012 Jun 13;13:131. doi: 10.1186/1471-2105-13-131 (PMC3433362; doi:10.1186/1471-2105-13-131)
Supplement: Additional file 1 — GlobalMIT+.zip — The GlobalMIT+ toolbox Implementation of the proposed algorithms in Matlab and C++, together with the user’s guide [15,18-21,23-27,29-31,45-50]. [file 1471-2105-13-131-S1.zip › GlobalMIT_2.Beta/Doc/ICONIP11.pdf]

Polynomial Time Algorithm for Learning Globally Optimal Dynamic Bayesian Network

Nguyen Xuan Vinh[1], Madhu Chetty[1], Ross Coppel[2], and Pramod P. Wangikar[3]

[1] Gippsland School of Information Technology, Monash University, Australia
[2] Department of Microbiology, Monash University, Australia
[3] Chemical Engineering Department, Indian Institute of Technology, Mumbai, India
{vinh.nguyen,madhu.chetty,Ross.Coppel}@monash.edu, wangikar@iitb.ac.in

Abstract. This paper is concerned with the problem of learning the globally optimal structure of a dynamic Bayesian network (DBN). We propose using a recently introduced information theoretic criterion named MIT (Mutual Information Test) for evaluating the goodness-of-fit of the DBN structure. MIT has been previously shown to be effective for learning static Bayesian network, yielding results competitive to other popular scoring metrics, such as BIC/MDL, K2 and BD, and the well-known constraint-based PC algorithm. This paper adapts MIT to the case of DBN. Using a modified variant of MIT, we show that learning the globally optimal DBN structure can be efficiently achieved in polynomial time.

Keywords: Dynamic Bayesian network, global optimization, gene regulatory network.

1 Introduction

Bayesian network (BN) is a central topic in machine learning, and has found numerous applications [8]. Two important disadvantages when applying the traditional static BN model to certain domain problems, such as gene regulatory network reconstruction in bioinformatics, are: *(i)* BN does not have a mechanism for exploiting the temporal aspect of time-series data; and *(ii)* BN does not allow the modeling of cyclic phenomena, such as feed back loops, which are prevalent in biological systems [13, 9]. These drawbacks have motivated the development of the so-called dynamic Bayesian network (DBN). The simplest model of this type is the first-order Markov stationary DBN, in which both the structure of the network and the parameters characterizing it are assumed to remain unchanged over time, as exemplified in Fig. 1a. In this model, the value of a random variable (RV) at time $t+1$ is assumed to depend only on the value of its parents at time t. DBN accounts for the temporal aspect of time-series data, in that an edge must always direct forward in time, and allows feedback loops (Fig. 1b). Since its inception, DBN has received particular interest, especially from the bioinformatics community [7, 13, 14, 12]. Recent works in the machine learning community have progressed to allow more flexible DBN models, such as

B.-L. Lu, L. Zhang, and J. Kwok (Eds.): ICONIP 2011, Part III, LNCS 7064, pp. 719–729, 2011.
© Springer-Verlag Berlin Heidelberg 2011

one with, either parameters [5], or both structure and parameters [10,4] changing over time. It is worth noting that more flexible models generally require more data to be learned accurately. In situations where training data are scarce, such as in microarray experiments where the data size can be as small as a couple of dozen samples, a simpler model such as the first-order Markov stationary DBN might be a more suitable choice.

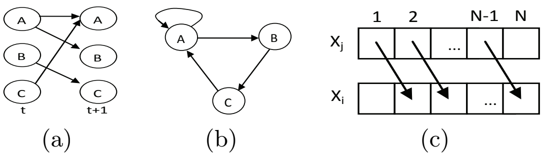

Fig. 1. Dynamic Bayesian Network: (a) 1st order Markov stationary DBN; (b) its equivalent folded network; (c) data alignment for dynamic Bayesian network with an edge $X_j \rightarrow X_i$. The "effective" number of observations is now only $N - 1$.

In this paper, we focus on the problem of learning the globally optimal structure for the first-order Markov stationary DBN. Henceforth, DBN shall refer to this particular class of stationary DBN, and *learning* shall refer to *structure learning*. The most popular approaches for learning DBN have been the ones adapted from the static BN literature, namely the *search+score* paradigm [13, 12], and Markov Chain Monte Carlo (MCMC) simulation [7, 4, 10]. In this paper, we are interested in the *search+score* approach, in which we specify a scoring function to assess the goodness-of-fit of a DBN given the data, and a search procedure to find the optimal network based on this scoring metric. Several popular scores for static BN, such as the Bayesian scores (K2, Bayesian-Dirichlet (BD), BDe and BDeu), and the information theoretic scores (Bayesian Information Criterion (BIC)/minimal description length (MDL) and Akaike Information Criterion—AIC), can be adapted straightforwardly for DBN. Another recently introduced scoring metric that catches our interest is the so-called MIT (Mutual Information Test) score [1], which, as the name suggests, belongs to the family of scores based on information theory. Through extensive experimental validation, the author suggests that MIT can compete favorably with Bayesian scores, outperforms BIC/MDL and should be the score of reference within those based on information theory. As opposed to the other popular scoring metrics, MIT has not been considered for DBN learning to our knowledge.

As for the *search* part, due to several non-encouraging complexity results (i.e., NP-hardness) in learning static BN [2], most authors have resorted to heuristic search algorithms when it comes to learning DBN. Recently, Dojer [3] has shown otherwise that learning DBN structure, as opposed to static BN, does not necessarily have to be NP-hard. In particular, this author showed that, under some mild assumptions, there are algorithms for finding the globally optimal network with a polynomial worst-case time complexity, when the MDL and BDe scores are used. In the same line of these findings, in this paper, we shall show

that there exists a polynomial worst-case time complexity algorithm for learning the globally optimal DBN under the newly introduced MIT scoring metric. Our experimental results show that, in terms of the recovered DBN quality, MIT performs competitively with BIC/MDL and BDe. In terms of theoretical complexity analysis, globalMIT admits a comparable worst-case complexity to the BIC/MDL-based global algorithm, and is much faster than the BDe-based algorithm. The paper is organized as follows: in Section 2 we review the MIT score for DBN learning. Section 3 presents our algorithm for finding the globally optimal network, followed by experimental results in section 4.

2 MIT Score for Dynamic Bayesian Network Learning

Let us first review the MIT score for learning BN, which can then be adapted to the DBN case. Briefly speaking, under MIT the goodness-of-fit of a network is measured by the total mutual information shared between each node and its parents, penalized by a term which quantifies the degree of statistical significance of this shared information. Let $\mathbf{X} = \{X_1, \dots, X_n\}$ denote the set of n variables with corresponding $\{r_1, \dots, r_n\}$ discrete states, D denote our data set of N observations, G be a DAG, and $\mathbf{Pa}_i = \{X_{i1}, \dots, X_{is_i}\}$ be the set of parents of X_i in G with corresponding $\{r_{i1}, \dots, r_{is_i}\}$ discrete states, $s_i = |\mathbf{Pa}_i|$, then the MIT score is defined as:

$$SS_{MIT}(G : D) = \sum_{i=1;\mathbf{Pa}_i \neq \emptyset}^{n} \{2N \cdot I(X_i, \mathbf{Pa}_i) - \sum_{j=1}^{s_i} \chi_{\alpha, l_{i\sigma_i(j)}}\}$$

where $I(X_i, \mathbf{Pa}_i)$ is the mutual information between X_i and its parents as estimated from D. $\chi_{\alpha,l_{ij}}$ is the value such that $p(\chi^2(l_{ij}) \le \chi_{\alpha,l_{ij}}) = \alpha$ (the Chi-square distribution at significance level $1-\alpha$), and the term $l_{i\sigma_i(j)}$ is defined as:

$$l_{i\sigma_i(j)} = \begin{cases} (r_i - 1)(r_{i\sigma_i(j)} - 1) \prod_{k=1}^{j-1} r_{i\sigma_i(k)}, & j = 2 \dots, s_i \\ (r_i - 1)(r_{i\sigma_i(j)} - 1), & j = 1 \end{cases}$$

where $\sigma_i = \{\sigma_i(1), \dots, \sigma_i(s_i)\}$ is any permutation of the index set $\{1 \dots s_i\}$ of $\mathbf{Pa}_i$, with the first variable having the greatest number of states, the second variable having the second largest number of states, and so on. It can be shown that the mutual information part of the score is equivalent to the log-likelihood score, while the second part serves as a penalty term. For detailed motivations and derivation of this scoring metric as well as an extensive comparison with BIC/MDL and BD, we refer readers to [1].

Adapting MIT for DBN learning is rather straightforward. Essentially, the mutual information is now calculated between a parent set and its child, which should be 1-unit shifted in time, as required by the first-order Markov assumption, denoted by $X_i^{\overrightarrow{1}} = \{X_{i2}, X_{i3}, \dots, X_{iN}\}$. As such, the number of "effective" observations, denoted by N_e, for DBN is now only $N-1$. Similarly, when the data is composed of N_t separate time-series, the number of effective observations is only $N_e = N - N_t$. This is demonstrated in Figure 1(c). The MIT score for DBN should be calculated as:

$$S'_{MIT}(G : D) = \sum_{i=1;\mathbf{Pa}_i \neq \emptyset}^{n} \{2N_e . I(X_i^{\overrightarrow{1}}, \mathbf{Pa}_i) - \sum_{j=1}^{s_i} \chi_{\alpha, l_i \sigma_i(j)}\}$$

3 Optimal Dynamic Bayesian Network Structure Learning in Polynomial Time with MIT

In this section, we show that learning the globally optimal DBN with MIT can be achieved in polynomial time. Our development is based on a recent result presented in [3], which states that under several mild assumptions, there exists a polynomial worst-case time complexity algorithm for learning the optimal DBN with the MDL and BDe scoring metrics. Specifically, the 4 assumptions that Dojer [3] considered are:

Assumption 1. *(acyclicity) There is no need to examine the acyclicity of the graph.*

Assumption 2. *(additivity)* $S(G : D) = \sum_{i=1}^{n} s(X_i, \boldsymbol{Pa}_i : D|_{X_i \cup \boldsymbol{Pa}_i})$ *where* $D|_{X_i \cup \boldsymbol{Pa}_i}$ *denotes the restriction of* D *to the values of the members of* $X_i \cup \boldsymbol{Pa}_i$.

To simplify notation, we write $s(\mathbf{Pa}_i)$ for $s(X_i, \mathbf{Pa}_i : D|_{X_i \cup \mathbf{Pa}_i})$.

Assumption 3. *(splitting)* $s(\boldsymbol{Pa}_i) = g(\boldsymbol{Pa}_i) + d(\boldsymbol{Pa}_i)$ *for some non-negative functions g,d satisfying* $\boldsymbol{Pa}_i \subseteq \boldsymbol{Pa}'_i \Rightarrow g(\boldsymbol{Pa}_i) \leq g(\boldsymbol{Pa}'_i)$

Assumption 4. *(uniformity)* $|\boldsymbol{Pa}_i| = |\boldsymbol{Pa}'_i| \Rightarrow g(\boldsymbol{Pa}_i) = g(\boldsymbol{Pa}'_i)$

Assumption 1 is valid for DBN in general (since the edges only directs forward in time, acyclicity is automatically satisfied). Assumption 2 states that the scoring function decomposes over the variables, which is obvious for MIT. Together with assumption 1, this assumption allows us to compute the parents set of each variable independently. Assumption 3 requires the scoring function to decompose into two components: d evaluating the accuracy of representing the distribution underlying the data by the network, and g measuring its complexity. Furthermore, g is required to be a monotonically non-decreasing function in the cardinality of $\mathbf{Pa}_i$ (assumption 4).

We note that unlike MIT in its original form that we have considered above, where better networks have higher scores, for the score considered by Dojer, lower scored networks are better. And thus the corresponding optimization must be cast as a score minimization problem. We now consider a variant of MIT as follows:

$$S_{MIT}(G : D) = \sum_{i=1}^{n} 2N_e.I(\overrightarrow{X_i^1}, \mathbf{X}) - S'_{MIT}(G : D) \quad (1)$$

which admits the following decomposition over each variable (with the convention of $I(X_i, \emptyset) = 0$):

$$s_{MIT}(\mathbf{Pa}_i) = d_{MIT}(\mathbf{Pa}_i) + g_{MIT}(\mathbf{Pa}_i)$$
$$d_{MIT}(\mathbf{Pa}_i) = 2N_e.I(\overrightarrow{X_i^1}, \mathbf{X}) - 2N_e.I(\overrightarrow{X_i^1}, \mathbf{Pa}_i)$$
$$g_{MIT}(\mathbf{Pa}_i) = \sum_{j=1}^{s_i} \chi_{\alpha, l_i\sigma_i(j)}$$

Roughly speaking, d_{MIT} measures the "error" of representing the joint distribution underlying D by G, while g_{MIT} measures the complexity of this representation. It is obvious that the problem of S'_{MIT} maximization is equivalent to the problem of S_{MIT} minimization, since $\sum_{i=1}^{n} 2N_e.I(\overrightarrow{X_i^1}, \mathbf{X}) = const.$ Also, it is straight-forward to show that d_{MIT} and g_{MIT} satisfy assumption 3. Unfortunately, g_{MIT} does not satisfy assumption 4. However, for many applications, if all the variables have the same number of states then it can be shown that g_{MIT} satisfies assumption 4.

Assumption 5. *(variable uniformity) All variables in* $\mathbf{X}$ *have the same number of discrete states* k*.*

Proposition 1. *Under the assumption of variable uniformity,* g_{MIT} *satisfies assumption 4.*

Proof. It can be seen that if $|\mathbf{Pa}_i| = |\mathbf{Pa}'_i| = s_i$, then $g_{MIT}(\mathbf{Pa}_i) = g_{MIT}(\mathbf{Pa}'_i) = \sum_{j=1}^{s_i} \chi_{\alpha,(k-1)^2 k^{j-1}}$. □

Since $g_{MIT}(\mathbf{Pa}_i)$ is the same for all parent sets of the same cardinality, we can write $g_{MIT}(|\mathbf{Pa}_i|)$ in place of $g_{MIT}(\mathbf{Pa}_i)$. With assumptions 1-5 satisfied, we can employ the following Algorithm 1, named globalMIT, to find the globally optimal DBN with MIT, i.e., the one with the minimal S_{MIT} score.

Algorithm 1. globalMIT : Optimal DBN with MIT

```
Pa_i := ∅
for p = 1 to n do
    If g_MIT(p) ≥ s_MIT(Pa_i) then return Pa_i; Stop.
    P = arg min_{Y⊆X:|Y|=p} s_MIT(Y)
    If s_MIT(P) < s_MIT(Pa_i) then Pa_i := P.
end for
```

Theorem 1. *Under assumptions 1-5, globalMIT applied to each variable in* $\mathbf{X}$ *finds a globally optimal DBN under the MIT scoring metric.*

Proof. The key insight here is that once a parent set grows to a certain extent, its complexity alone surpasses the total score of a previously found sub-optimal parent set. In fact, all the remaining potential parent sets $\mathbf{P}$ omitted by the algorithm have a total score higher than the current best score, i.e., $s_{MIT}(\mathbf{P}) \geq g_{MIT}(|\mathbf{P}|) \geq s_{MIT}(\mathbf{Pa}_i)$, where $\mathbf{Pa}_i$ is the last sub-optimal parent set found. □

We note that the terms $2N_e.I(\overrightarrow{X_i^1}, \mathbf{X})$ in the S_{MIT} score in (1) do not play any essential role, since they are all constant and would not affect the outcome of our optimization problem. Knowing their exact value is however, necessary for the stopping criterion in Algorithm 1, and also for constructing its complexity bound, as we shall do shortly. Unfortunately, calculating $I(\overrightarrow{X_i^1}, \mathbf{X})$ is by itself a hard problem, requiring $O(k^{n+1})$ space and time in general. However, for our purpose, since the only requirement for d_{MIT} is that it must be non-negative,

it is sufficient to use an upper bound of $I(\overrightarrow{X_i^1}, \mathbf{X})$. A fundamental property of the mutual information states that $I(\mathbf{X}, \mathbf{Y}) \leq \min\{H(\mathbf{X}), H(\mathbf{Y})\}$, i.e., mutual information is bounded by the corresponding entropies. We therefore have:

$$2N_e.I(\overrightarrow{X_i^1}, \mathbf{X}) \leq 2N_e.H(\overrightarrow{X_i^1}),$$

where $H(\overrightarrow{X_i^1})$ can be estimated straightforwardly from the data. Or else, we can use an a priory fixed upper bound for all $H(\overrightarrow{X_i^1})$, that is $\log k$, then:

$$2N_e.I(\overrightarrow{X_i^1}, \mathbf{X}) \leq 2N_e.\log k.$$

Using these bounds, we obtain the following more practical versions of d_{MIT}:

$$d'_{MIT}(\mathbf{Pa}_i) = 2N_e.H(\overrightarrow{X_i^1}) - 2N_e.I(\overrightarrow{X_i^1}, \mathbf{Pa}_i)$$
$$d''_{MIT}(\mathbf{Pa}_i) = 2N_e.\log k - 2N_e.I(\overrightarrow{X_i^1}, \mathbf{Pa}_i)$$

It is straightforward to show that Algorithm 1 and Theorem 1 are still valid when d'_{MIT} or d''_{MIT} are used in place of d_{MIT}.

3.1 Complexity Bound

Theorem 2. *globalMIT admits a polynomial worst-case time complexity in the number of variables.*

Proof. Our aim is to find a number p^* satisfying $g_{MIT}(p^*) \geq s_{MIT}(\emptyset)$. Clearly, there is no need to examine any parent set of cardinality p^* and over. In the worse case, our algorithm will have to examine all the possible parent sets of cardinality from 1 to $p^* - 1$. We have:

$$g_{MIT}(p^*) \geq s_{MIT}(\emptyset) \Leftrightarrow \sum_{j=1}^{p^*} \chi_{\alpha, l_i \sigma_i(j)} \geq d_{MIT}(\emptyset) = 2N_e.I(\overrightarrow{X_i^1}, \mathbf{X}).$$

As discussed above, since calculating d_{MIT} is not convenient, we use d'_{MIT} and d''_{MIT} instead. With d'_{MIT} and d''_{MIT}, p^* can be found respectively as:

$$p^* = \arg\min\{p | \textstyle\sum_{j=1}^{p} \chi_{\alpha, l_i \sigma_i(j)} \geq 2N_e.H(\overrightarrow{X_i^1})\} \quad (2)$$
$$p^* = \arg\min\{p | \textstyle\sum_{j=1}^{p} \chi_{\alpha, l_i \sigma_i(j)} \geq 2N_e.\log k\}. \quad (3)$$

It can be seen that p^* depends only on α, k and N_e. Since there are $O(n^{p^*})$ subsets with at most p^* parents, and each set of parents can be scored in polynomial time, globalMIT admits an overall polynomial worst-case time complexity in the number of variable n. □

We now give some examples to demonstrate the practicability of Theorem 2.

Example 1: Consider a gene regulatory network reconstruction problem, where each gene has been discretized to $k = 3$ states, corresponding to up, down and regular gene expression. With the level of significance α set to 0.999 as recommended in [1], we have $g_{MIT}(1) = 18.47$; $g_{MIT}(2) = 51.37$; $g_{MIT}(3) = 119.35\ldots$ Consider a data set of $N = 12$ observations, which is the popular length of microarray time-series experiments (in fact N often ranges within $4-15$), then $d''_{MIT}(\emptyset) = 2(N-1)\log k = 24.16$. Observing that $g_{MIT}(2) > d''_{MIT}(\emptyset)$, then $p^* = 2$ and we do not have to consider any parent sets of 2 variables or more. Let us compare this bound with those of the algorithms for learning the globally optimal DBN under the BIC/MDL and BDe scoring metrics. For BIC/MDL, p^*_{MDL} is given by $\lceil \log_k N \rceil$, while for BDe, $p^*_{BDe} = \lceil N \log_{\gamma^{-1}} k \rceil$, where the distribution $P(G) \propto \gamma^{\sum |\mathbf{Pa}_i|}$, with a penalty parameter $0 < \gamma < 1$, is used as a prior over the network structures [3]. In this case, $p^*_{MDL} = 3$. If we choose $\log \gamma^{-1} = 1$ then $p^*_{BDe} = \lceil N \log k \rceil = 14$. In general, p^*_{BDe} scales linearly with the number of data items N, making its value less of practical interest, even for small data sets.

Example 2: Since the number of observations in a single microarray time-series experiment is often limited, it is a popular practice to concatenate several time-series to obtain a larger data set for analysis. Let us merge $N_t = 10$ data sets, each with 12 observations, then $N_e = N - N_t = 120 - 10 = 110$. For this combined data set, $g_{MIT}(4) > d''_{MIT}(\emptyset) = 2N_e \log k = 241.69 \Rightarrow p^* = 4$, thus there is no need to consider any parent set of more than 3 variables. Of course, this analysis only gives us the worst-case time complexity. In practice, the execution of Algorithm 1 can often be much shorter, since $s_{MIT}(\mathbf{Pa}_i)$ is often much greater than $s_{MIT}(\emptyset)$. For comparison, we have $p^*_{MDL} = 5$, and $p^*_{BDe} = 132$ with $\log \gamma^{-1} = 1$.

4 Experimental Evaluation

We next describe our experiments to evaluate our global approach for learning DBN with MIT, and compare it with the other most popular scores, namely BIC/MDL and BD. Our method, implemented in Matlab, was used, along with BNFinder [12], a Python-based software for inferring the globally optimal DBN with the MDL and BDe scores as proposed by Dojer [3]. In addition, we also employed the Java-based Banjo software [6], which can perform greedy search and simulated annealing over the DBN space using the BDeu metric. The specific problem domain that we shall work with in this experiment is the problem of gene regulatory network reconstruction from microarray data, with the variables being genes, and edges being regulatory relationship between genes. We employ several synthetic data sets generated by different data generation schemes that have been used in some previous studies, namely, probabilistic method [7], linear dynamical system based method [13], and non-linear dynamical system based method [11]. As a realistic number of samples for microarray data, we generated data sets of between 30 and 300 samples. With the ground-truth network available, we count the number of true positive (TP), false positive (FP), true negative (TN)

and false negative (FN) edges, and report two network quality metrics, namely *sensitivity*= TP/(TP+FN), and *imprecision*=FP/(FP+TP).

Parameters setting: globalMIT has one parameter, namely the significance level α, to control the trade-off between goodness-of-fit and network complexity. Adjusting α will generally affect the sensitivity and imprecision of the discovered network, very much like its affect on the Type-I and Type-II error of the mutual information test of independence. de Campos [1] suggested using very high levels of significance, namely 0.999 and 0.9999. We note that, the data sets used in [1] are of sizes from 1000 to 10000 samples. For microarray data sets of merely $30-300$ samples, it is necessary to use a lower level of significance α to avoid overly penalizing network complexity. We have experimentally observed that using $\alpha \in [0.95, 0.999]$ on these small data sets yielded reasonable results, with balanced sensitivity and imprecision. BNFinder+MDL required no parameter tuning, while for BNFinder+BDe, the pseudo-counts for the BDe score was set to the default value of 1, and the penalty parameter was set to the default value of $\log \gamma^{-1} = 1$. For Banjo, we employed simulated annealing as the search engine, and left the equivalent sample size to the default value of 1 for the BDeu score, while the max-fan-in was set to 3. The runtime for Banjo was set to the average runtime of globalMIT, with a minimum value of 10 minutes, in case where globalMIT terminates earlier. Since some experiments were time consuming, all our experiments were performed in parallel on a 16-core Xeon X5550 workstation.

Probabilistic Network Synthetic Data: We employed a subnetwork of the yeast cell cycle, consisting of 12 genes and 11 interactions, as depicted in Fig. 2(a). Two different conditional probabilities were associated with these interactions, namely noisy regulation according to a binomial distribution, and noisy XOR-style co-regulation (see [7] for the parameter details, and this author website for Matlab code to generate this data). In addition, 8 unconnected nodes were added as confounders, for a total of 20 nodes. For each number of samples $N = 30, 70$ and 100, we generated 10 data sets. From the average statistics in Table 1, it can be seen that this is a relatively easy case for all methods. Except Banjo which committed a lower sensitivity and yet a higher imprecision, all other methods nearly recovered the correct network. Note that due to the excessive runtime of BNFinder+BDe, for $N = 100$, only 1 of ten data sets was analyzed.

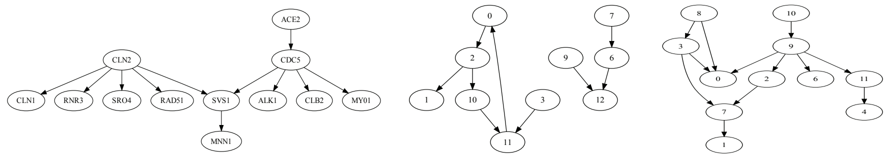

(a) The yeast cell cycle (b) Yu's net No. 1 (c) Yu's net No. 2

Fig. 2. Synthetic Dynamic Bayesian Networks

Linear Dynamical System Synthetic Data: We employed the two synthetic networks as described in [13], each consisting of 20 genes, with 10 and 11 genes having regulatory interaction, while the remainder moving in a random walk, as depicted in Fig. 2(b,c). The data are generated by a simple linear dynamical process as: $X_{t+1} - X_t = A(X_t - T) + \epsilon$, with X denoting the expression profiles, A describes the strength of gene-gene regulations, T is the constitutive expression values, and ϵ simulates a uniform biological noise. The detailed parameters for each network can be found in [13]. Using the GeneSim software provided by these authors, we generated, for each number of sample $N = 100, 200$ and 300, 10 data sets for each network. From the average statistics in Table 1, it can be seen that Banjo performed well on both data sets. BNFinder achieved a slightly lower sensitivity, but with very high imprecision rate. It is probable that the self-link suppression default option in BNFinder has led the method to include more incorrect edges to the network for a reasonable goodness-of-fit. GlobalMIT performed worse at $N = 100$, but is better at higher number of samples. Again, due to time limit, we were only able to run BNFinder+BDe on one out of ten data sets for each network at $N = 200$ and 300.

Table 1. Experimental Results

N	GlobalMIT			Banjo			BNFinder+MDL			BNFinder+BDe		
	Sen	Imp	Time	Sen	Imp	Time	Sen	Imp	Time	Sen	Imp	Time
Probabilistic Network Synthetic Data												
30	95 ± 9	29 ± 13	13 ± 3	84 ± 6	70 ± 4	600	86 ± 10	10 ± 9	< 2	85 ± 8	11 ± 11	52 ± 4
70	100 ± 0	1 ± 3	67 ± 4	82 ± 0	51 ± 6	600	100 ± 0	5 ± 7	25 ± 1	100 ± 0	3 ± 4	2.7 ± 0.5h
100	100 ± 0	0 ± 0	499 ± 56	82 ± 0	43 ± 2	600	100 ± 0	1 ± 3	34 ± 1	100	0	9.4h*
Linear Dynamical System Synthetic Data: Yu's net No. 1												
100	54 ± 12	54 ± 13	66 ± 5	58 ± 9	35 ± 16	600	58 ± 9	72 ± 4	4 ± 1	67 ± 7	74 ± 4	4.4 ± 1.3h
200	77 ± 4	19 ± 9	409 ± 127	67 ± 5	8 ± 9	600	66 ± 4	74 ± 2	47 ± 5	67	84	13.6h*
300	79 ± 4	19 ± 12	$.6 \pm .07$h	69 ± 7	4 ± 6	0.6h	68 ± 4	77 ± 2	49 ± 5	67	84	26.5h*
Linear Dynamical System Synthetic Data: Yu's net No. 2												
100	22 ± 15	72 ± 17	44 ± 8	38 ± 11	59 ± 13	600	28 ± 12	83 ± 7	3 ± 1	30 ± 16	86 ± 7	3.3 ± 0.8h
200	49 ± 15	35 ± 19	534 ± 158	45 ± 14	37 ± 16	600	38 ± 8	79 ± 4	39 ± 5	42	85	12.1h*
300	62 ± 12	24 ± 11	$.49 \pm .05$h	53 ± 9	17 ± 13	0.49h	47 ± 9	78 ± 4	40 ± 6	50	85	21.2h*
Non-Linear Dynamical System Synthetic Data												
99	37 ± 10	59 ± 12	< 1	7 ± 3	13 ± 32	600	13 ± 11	81 ± 14	< 1	16 ± 13	77 ± 17	< 1
150	39 ± 16	58 ± 16	< 1	9 ± 11	16 ± 35	600	19 ± 15	71 ± 23	< 1	24 ± 18	67 ± 24	< 1
300	61 ± 7	51 ± 6	< 1	10 ± 12	30 ± 48	600	24 ± 14	74 ± 14	< 1	23 ± 20	80 ± 15	< 1

Sen: percent sensitivity; Imp: percent imprecision; Time: in seconds, unless otherwise specified
*: only run on one data set.

Non-Linear Dynamical System Synthetic Data: We employed a five-gene network as in [11], of which dynamics is modeled by a system of coupled differential equations adhering to the power-law formalism, called the S-system. The concrete form of an S-system is given as follows:

$$\frac{dX_i}{dt} = \alpha_i \prod_{j=1}^{n} X_j^{g_{ij}} - \beta_i \prod_{j=1}^{n} X_j^{h_{ij}}, \quad i = 1 \dots n, \tag{4}$$

where the rates α_i, β_i and kinetic orders g_{ij} and h_{ij} are parameters dictating the influence of gene X_j on the rate of change in the expression level of gene

X_i. Using the same system parameters as in [11], we integrated the system using the Runge-Kutta method with 10 different initial conditions to obtain 10 time series, each of length 50. We then randomly chose 3 time series of length 33, 3 of length 50 and 6 of length 50, to make data sets of length $N = 99, 150$ and 300 respectively, with 10 data sets for each N value. Although this data had been previously analyzed with good accuracy by using differential equation models, it proved to be the most challenging case for DBN based methods. Even with a fairly large number of samples, compared to a small number of variables and interactions, all the methods performed poorly, with low sensitivity and high imprecision, rendering the results hardly useful. GlobalMIT nevertheless showed a slight advantage, with a reasonable sensitivity and imprecision at $N = 300$.

5 Conclusion

This paper has investigated the problem of learning the globally optimal DBN structure with the MIT scoring metric. We have showed that this task can be achieved using a polynomial time algorithm. Compared with the other well-known scoring metrics, namely BIC/MDL and BDe, both in terms of the worst-case complexity bound and practical evaluation, the BIC/MDL-based algorithm for learning the globally optimal DBN is fastest, followed by MIT, whereas the extensive runtime required by the BDe-based algorithm renders it a very expensive option. GlobalMIT, which is based on a sound information theoretic criterion, represents a very competitive alternative, both in terms of the network quality and runtime required.

Acknowledgments. This project is supported by an Australia-India strategic research fund (AISRF). Implementation of the proposed algorithms in Matlab and C++ is available at http://code.google.com/p/globalmit.

References

1. de Campos, L.M.: A scoring function for learning bayesian networks based on mutual information and conditional independence tests. J. Mach. Learn. Res. 7, 2149–2187 (2006)
2. Chickering, D.M.: Learning Bayesian Networks is NP-Complete. In: Fisher, D., Lenz, H. (eds.) Learning from Data: Artificial Intelligence and Statistics V, pp. 121–130 (1996)
3. Dojer, N.: Learning Bayesian Networks Does Not Have to Be NP-Hard. In: Proceedings of International Symposium on Mathematical Foundations of Computer Science, pp. 305–314 (2006)
4. Dondelinger, F., Lebre, S., Husmeier, D.: Heterogeneous continuous dynamic bayesian networks with flexible structure and inter-time segment information sharing. In: ICML, pp. 303–310 (2010)
5. Grzegorczyk, M., Husmeier, D.: Non-stationary continuous dynamic Bayesian networks. In: NIPS 2009 (2009)

6. Hartemink, A.: Banjo: A structure learner for static and dynamic bayesian networks, http://www.cs.duke.edu/~amink/software/banjo
7. Husmeier, D.: Sensitivity and specificity of inferring genetic regulatory interactions from microarray experiments with dynamic Bayesian networks. Bioinformatics 19(17), 2271–2282 (2003)
8. Koller, D., Friedman, N.: Probabilistic Graphical Models: Principles and Techniques. The MIT Press (2009)
9. Ram, R., Chetty, M., Dix, T.: Causal modeling of gene regulatory network. In: IEEE CIBCB 2006 (2006)
10. Robinson, J., Hartemink, A.: Learning Non-Stationary Dynamic Bayesian Networks. The Journal of Machine Learning Research 11, 3647–3680 (2010)
11. Sugimoto, N., Iba, H.: Inference of gene regulatory networks by means of dynamic differential bayesian networks and nonparametric regression. Genome Informatics 15(2), 121–130 (2004)
12. Wilczynski, B., Dojer, N.: BNFinder: exact and efficient method for learning Bayesian networks. Bioinformatics 25(2), 286–287 (2009)
13. Yu, J., Smith, V.A., Wang, P.P., Hartemink, A.J., Jarvis, E.D.: Advances to Bayesian network inference for generating causal networks from observational biological data. Bioinformatics 20(18), 3594–3603 (2004)
14. Zou, M., Conzen, S.D.: A new dynamic Bayesian network (DBN) approach for identifying gene regulatory networks from time course microarray data. Bioinformatics 21(1), 71–79 (2005)
