# Supplementary figures and images for: Gene regulatory network modeling via global optimization of high-order dynamic Bayesian network
Source: BMC Bioinformatics. 2012 Jun 13;13:131. doi: 10.1186/1471-2105-13-131 (PMC3433362; doi:10.1186/1471-2105-13-131)

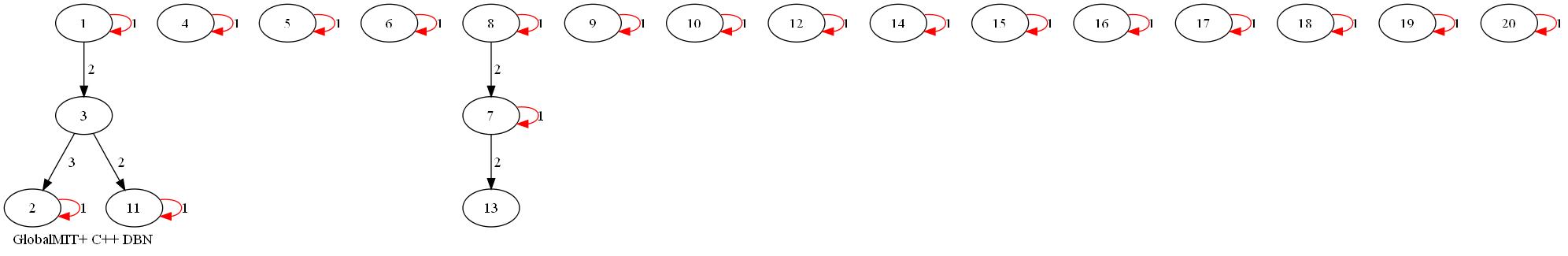

Supplement: Additional file 1 — GlobalMIT+.zip — The GlobalMIT+ toolbox Implementation of the proposed algorithms in Matlab and C++, together with the user’s guide [15,18-21,23-27,29-31,45-50]. [file 1471-2105-13-131-S1.zip › GlobalMIT_2.Beta/myDefaultGraph.jpg]

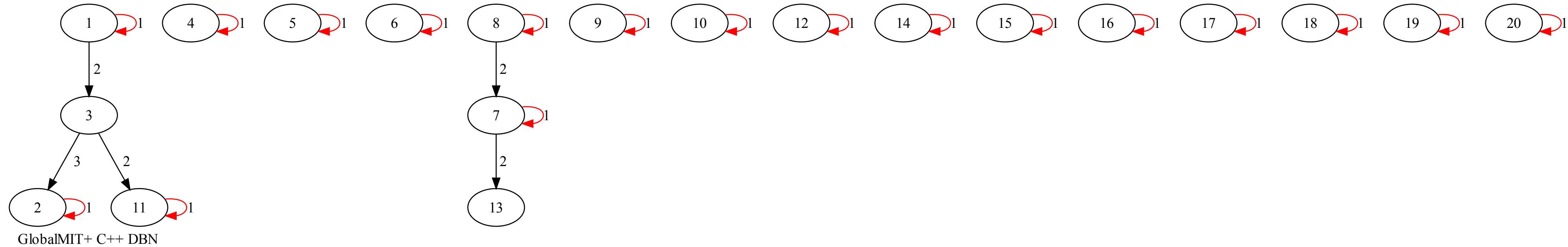

Supplement: Additional file 1 — GlobalMIT+.zip — The GlobalMIT+ toolbox Implementation of the proposed algorithms in Matlab and C++, together with the user’s guide [15,18-21,23-27,29-31,45-50]. [file 1471-2105-13-131-S1.zip › GlobalMIT_2.Beta/myDefaultGraph.pdf]
